# The safety, physiological response and repeatability of the incremental shuttle walk test in survivors of COVID-19

**DOI:** 10.1183/23120541.00089-2025

**Published:** 2025-12-15

**Authors:** Molly M. Baldwin, Enya Daynes, Urvee Karsanji, Hamish J.C. McAuley, Nicolette C. Bishop, Charlotte E. Bolton, William D-C. Man, Ioannis Vogiatzis, James D. Chalmers, Ling-Pei Ho, Alex Horsley, Michael Marks, Krisnah Poinasamy, Betty Raman, Olivia C. Leavy, Matthew Richardson, Omer Elneima, Aarti Shikotra, Amisha Singapuri, Marco Sereno, Ruth M. Saunders, Victoria C. Harris, Linzy Houchen-Wolloff, Neil J. Greening, Ewen M. Harrison, Annemarie B. Docherty, Nazir I. Lone, Jennifer K. Quint, Louise V. Wain, Christopher E. Brightling, Rachael A. Evans, Sally J. Singh

**Affiliations:** 1 NIHR Leicester Biomedical Research Centre, Leicester, UK; 2 Department of Respiratory Sciences, University of Leicester, Leicester, UK; 3 Centre for Exercise and Rehabilitation Science, University Hospitals of Leicester NHS Trust, Leicester, UK; 4 National Centre for Sport and Exercise Medicine, School of Sport, Exercise and Health Sciences, Loughborough University, Loughborough, UK; 5 Centre for Respiratory Research, Translational Medical Sciences, School of Medicine, University of Nottingham, Nottingham, UK; 6 NIHR Nottingham Biomedical Research Centre, Nottingham, UK; 7 Harefield Respiratory Research Group, Royal Brompton and Harefield Hospitals, Guy's and St Thomas’ NHS Foundation Trust, London, UK; 8 National Heart and Lung Institute, Imperial College London, London, UK; 9 Department of Sport, Exercise and Rehabilitation, Faculty of Health and Life Sciences, Northumbria University, Newcastle, UK; 10 Newcastle upon Tyne Hospitals NHS Foundation Trust, Translational and Clinical Research Institute, Newcastle University, Newcastle, UK; 11 University of Dundee, Ninewells Hospital and Medical School, Dundee, UK; 12 MRC Translational Immune Discovery Unit, University of Oxford, Oxford, UK; 13 NIHR Oxford Biomedical Research Centre, Oxford, UK; 14 NIHR Manchester Biomedical Research Centre, Manchester, UK; 15 Manchester University NHS Foundation Trust, Manchester, UK; 16 Department of Clinical Research, London School of Hygiene and Tropical Medicine, London, UK; 17 Hospital for Tropical Diseases, University College London Hospital, London, UK; 18 Asthma+Lung UK, London, UK; 19 Radcliffe Department of Medicine, University of Oxford, Oxford, UK; 20 Oxford University Hospitals NHS Foundation Trust, Oxford, UK; 21 Department of Population Health Sciences, University of Leicester, Leicester, UK; 22 The Usher Institute, University of Edinburgh, Edinburgh, UK; 23 School of Public Health, Imperial College London, London, UK; 24 These authors contributed equally

## Abstract

**Background:**

The incremental shuttle walk test (ISWT) may be a valuable tool for measuring exercise tolerance in patients after a hospital admission with COVID-19. However, the safety, physiological response and repeatability of the ISWT are unknown in this cohort. The present study aimed to explore the properties of this test using the Post-Hospital COVID-19 (PHOSP-COVID) study.

**Methods:**

Participants performed two ISWTs, with a 30-min rest between tests, at 5 and 12 months post-hospital discharge for COVID-19. Heart rate and fingertip peripheral oxygen saturation were recorded pre- and post-test. Reasons for test termination were noted.

**Results:**

1593 individuals (median (interquartile range) age 58 (50–66) years and body mass index 31.2 (27.6–35.8) kg·m^−^^2^; 967 (60.7%) males) performed an ISWT; two tests were performed by 1034 and 390 participants at the 5- and 12-month visit, respectively. At 5 months post-discharge, six patients (0.4%) had an adverse event and the most common reason contributing to test termination was breathlessness (826 (54.2%) participants). 336/1470 (22.9%) participants experienced exertional desaturation. Distance walked was greater in the second ISWT compared to the first ISWT at 5 and 12 months post-discharge (mean±sd difference: 5 months: 19±94 m; 12 months: 11±80 m; p<0.05), with an intraclass correlation coefficient estimate of 0.96 (95% CI 0.95–0.97) at 5 months and 0.97 (95% CI 0.96–0.97) at 12 months.

**Conclusions:**

The ISWT appeared to be safe in this large cohort, supporting use of this field walking test for this population in clinical and research settings. A familiarisation test is recommended, with further study needed to determine the number of familiarisation tests required to achieve acceptable within-day repeatability.

## Shareable abstract

**The incremental shuttle walk test appears to be safe in individuals following a COVID-19 hospitalisation, supporting use of this field walking test for this population in clinical and research settings. A familiarisation walk is recommended in this cohort.** https://bit.ly/42QpZQ2

## Introduction

After a hospital admission due to severe acute respiratory syndrome coronavirus 2 (SARS-CoV-2) infection many individuals experience persistent symptoms after their initial infection has resolved, with the exact prevalence reported influenced by the time frame used, population, vaccination status and symptoms investigated [1–5]. The most commonly reported symptoms, including fatigue and breathlessness, may be related to the underlying disease or hospital treatment received, such as mechanical ventilation, sedation or prolonged physical inactivity [6, 7]. These symptoms contribute to reduced exercise tolerance in chronic respiratory and cardiac diseases, and are targeted by rehabilitation strategies [8, 9]. Measuring exercise tolerance in individuals after a COVID-19 hospitalisation is of clinical importance to determine abnormal exercise capacity and assess the effectiveness of rehabilitation interventions. The latter has been identified as a research priority by individuals with ongoing symptoms of COVID-19, carers, clinicians and clinical researchers [10].

A ramp-incremental exercise test to symptom limitation, combined with comprehensive breath-by-breath monitoring of cardiopulmonary gas exchange variables, is the gold standard for measuring exercise capacity [11]. However, the special equipment, trained personnel and time needed to perform cardiopulmonary exercise tests can be difficult to fulfil, preventing widespread use in hospitals and rehabilitation centres. The unknown risk of SARS-CoV-2 transmission during these tests also limited availability further during the pandemic.

The incremental shuttle walk test (ISWT) is a field-based walking test often employed to measure exercise tolerance in clinical settings, as it is a maximal exercise test that is standardised, requires little equipment and training, and is low cost. Importantly, the ISWT induces cardiorespiratory responses that are similar to those seen in ramp-incremental exercise tests and can be performed on a shorter track than the 6-min walk test (6MWT) (10 *versus* 30 m), enabling more centres to house the test [12]. Alongside these practicalities, the ISWT has been shown to be a valid measure of exercise tolerance that is repeatable after a familiarisation test in multiple health conditions [12–17]. Due to these characteristics, the ISWT is responsive to interventions and is commonly used as an outcome measure for clinical interventions, including rehabilitation programmes [18, 19]. The safety (adverse events and reasons for test termination) and physiological responses to the ISWT, including the prevalence of exertional desaturation, are unknown in the post-hospital COVID-19 population. It is also unknown whether a familiarisation test is needed in this cohort and if this can be performed on the same day. This is an exploratory analysis of the Post-Hospital COVID-19 (PHOSP-COVID) study that aims to assess the safety, physiological response and repeatability of the ISWT in individuals who have been discharged from hospital due to COVID-19.

## Methods

The PHOSP-COVID study design, rationale and a description of the cohort profile have been published previously [5, 20, 21]. In brief, PHOSP-COVID was a multicentre long-term follow-up study for adults (aged ≥18 years) discharged from a National Health Service hospital in the UK between 1 February 2020 and 31 March 2021, with laboratory- or clinician-confirmed COVID-19. All participants provided written informed consent. The study was approved by the Leeds West Research Ethics Committee (reference: 20/YH/0225) and is registered at the ISRCTN registry (ISRCTN10980107).

Exercise tolerance was assessed using the ISWT at research visits performed 5 and 12 months post-hospital discharge for COVID-19. The ISWT is an externally paced, incremental exercise test that requires the participant to walk around a 10-m course at a speed dictated by audio. The walking speed progressively increases each minute and the test is terminated when the participant is no longer able to keep up with the target walking speed [22]. The modified version of the ISWT was used that has 15 increment levels, affording a maximum distance of 1500 m. All ISWTs were performed in accordance with technical standards [19, 22]. Blood pressure, heart rate and fingertip peripheral oxygen saturation (*S*_pO_2__) were measured pre- and post-test whilst participants were sitting. Heart rate and fingertip *S*_pO_2__ were also monitored throughout the test using pulse oximetry. Exertional desaturation was defined as a pre- to post-test fall in *S*_pO_2__ ≥4% or a post-test *S*_pO_2__ <90% [23, 24]. Subjective ratings of leg fatigue and breathlessness were measured immediately pre- and post-test using the modified Borg scale [25]. Participants were asked if their reason for intolerance was 1) breathlessness, 2) leg fatigue, 3) breathlessness and leg fatigue or 4) other. Participants could provide multiple responses to this question. Adverse events, defined as any untoward medical occurrence relating to the ISWT or clinical investigation that has occurred from the ISWT, were reported.

Participants were then asked to perform a second ISWT at their research visit 5 months post-hospital discharge under the same conditions after a 30-min rest. If participants performed two tests at the 5-month visit, data from the ISWT with the greatest distance was included in the analysis characterising the physiological response to the test and reason(s) for test termination. Data from both walking tests were used to assess ISWT repeatability at 5 months post-discharge.

Some participants who performed two ISWTs at 5 months post-discharge attended the 12-month research visit and completed a further two ISWTs. This allowed a comparison between ISWT repeatability at 5 and 12 months post-hospital discharge. Within visit test–retest variability was calculated using the following equation: (standard error (sd/√2)/joint mean)×100.

### Statistical analysis

The Shapiro–Wilk test was used to determine the distribution of the data. Non-parametric data were expressed as median and interquartile range (IQR). Categorical variables were expressed as frequency and percentage. No data were imputed. Physiological measures recorded pre- and post-ISWT were compared using a Wilcoxon signed-rank test. An unpaired t-test and Chi-squared test were used to compare ISWT distance and the presence of a pre-existing respiratory comorbidity in those with and without exertional desaturation, respectively.

A paired t-test was used to compare the distance achieved in the first and second ISWT performed 5 months post-hospital discharge. A two-way repeated measures ANOVA with one within factor (test number: ISWT1 *versus* ISWT2) and one between factor (time: 5 *versus* 12 months) was used to assess differences over time between the distance achieved in ISWT1 and ISWT2. Bland–Altman plots were created to determine the degree of agreement between the distance achieved in ISWT1 and ISWT2 at both time-points [26]. The Breusch–Pagan test was used to test for heteroscedasticity in a linear regression on the absolute difference between the first and second ISWT *versus* the mean distance achieved. This test was performed to determine whether the variability in the difference between the first and second ISWT was dependent on the magnitude of mean ISWT distance. The coefficient of repeatability was calculated by multiplying the within-subject standard deviation by 2.77 (√2×1.96). An intraclass correlation coefficient (ICC) estimate and 95% confidence intervals were calculated to determine the repeatability of the distance walked in the ISWT. The ICC was calculated based on a mean rating (k=2), absolute agreement, two-way mixed effects model. The following ICC interpretation was used: poor agreement <0.5, moderate agreement 0.5–0.75, good agreement 0.76–0.9 and excellent agreement >0.9 [27]. Statistical significance was set at p<0.05. All analyses were performed using SPSS Statistics version 28 (IBM, Armonk, NY, USA) within the Public Health Scotland Trusted Research Environment.

## Results

1593 participants (table 1) from 25/36 (69.4%) Tier 2 study sites performed an ISWT 5 months post-discharge (median (IQR) 5.4 (4.0–6.3) months post-discharge) [5, 21]. Adverse events relating to the ISWT were reported for 6/1593 (0.4%) participants, with two participants requiring medical treatment; 8/1593 (0.5%) participants achieved the maximum distance of 1500 m [28]. The most commonly reported reasons for test termination were: breathlessness (826 (54.2%)), walking speed (474 (31.1%)), leg fatigue (445 (29.2%)), musculoskeletal pain (54 (3.5%)), other unreported reason (22 (1.4%)), dizziness (18 (1.2%)), test terminated by operator due to reduction in *S*_pO_2__ (14 (0.9%)) and general fatigue (11 (0.7%)).

**TABLE 1:** Participant characteristics

**Participants**	1593
**Age at admission (years)**	58 (50–66)
**Sex**	
Female	625 (39.2)
Male	967 (60.7)
Missing	1 (0.1)
**Ethnicity**	
White	1204 (75.6)
South Asian	195 (12.2)
Black	104 (6.5)
Mixed	33 (2.1)
Other	49 (3.1)
Missing	8 (0.5)
**BMI (kg·m^−2^)**	31.2 (27.6–35.8)
<30 kg·m^−2^	473 (29.7)
≥30 kg·m^−2^	655 (41.1)
Missing	465 (29.2)
**Fat-free mass (kg)**	58.5±14.2
**Comorbidities (n)**	2 (0.0–3.0)
0	399 (25.0)
1	368 (23.1)
≥2	826 (51.9)
**Length of admission (days)**	8 (4–14)
**Mechanical ventilation**	214 (13.4)
**ISWT**	
ISWT distance (m)	436±266
Walking aid	
Yes	77 (4.8)
No	1507 (94.6)
Missing	9 (0.6)
Supplementary oxygen	
Yes	14 (0.9)
No	1555 (97.6)
Missing	24 (1.5)
**Do you feel fully recovered from COVID-19?**	
Yes	330 (20.7)
No	759 (47.6)
Unsure	252 (15.8)
Missing	252 (15.8)

Data are presented as n, median (interquartile range), n (%) or mean±sd. BMI: body mass index; ISWT: incremental shuttle walk test.

### Physiological response to ISWT

Physiological measures recorded pre- and post-ISWT were available for 1470/1593 (92.3%) participants at the research visit conducted 5 months post-discharge (table 2). Participants achieved a mean±sd distance of 441±270 m. Heart rate, systolic blood pressure and diastolic blood pressure increased pre- to post-test by a median (IQR) of 25 (10–45) beats·min^−1^, 14 (3–28) mmHg and 2 (−3–7) mmHg, respectively (p<0.05) (table 2). Median (IQR) post-test heart rate (104 (85–125) beats·min^−1^) was less than median age-based predicted maximum heart rate (220−age (years); 162 (154–170) beats·min^−1^; p=0.01). 206/1470 (14.0%) participants achieved a post-test heart rate ≥85% predicted maximum. The median (IQR) change in *S*_pO_2__ pre- to post-test was −1% (−3–0%) (p=0.01). Ratings of Borg leg fatigue and breathlessness recorded at test termination were greater than pre-test values (p<0.05) (table 2).

**TABLE 2:** Physiological responses to incremental shuttle walk test (n=1470)

	Pre-test	Post-test
**Heart rate (beats·min^−1^)^#^**	76 (68–86)	104 (85–125)
**Fingertip *S*_pO_2__** **(%)^#^**	97 (96–98)	96 (94–98)
**Systolic blood pressure (mmHg)^#^**	135 (124–148)	152 (137–169)
**Diastolic blood pressure (mmHg)^#^**	83 (76–89)	84 (76–92)
**Borg breathlessness scale^#^**	0 (0–1)	3 (2–5)
**Borg leg fatigue scale^#^**	0 (0–1)	3 (1–4)

Data are presented as median (interquartile range). *S*_pO_2__: peripheral oxygen saturation. ^#^: statistically significant difference between pre- and post-test values (p<0.05).

### Exertional desaturation

**FIGURE 1:**
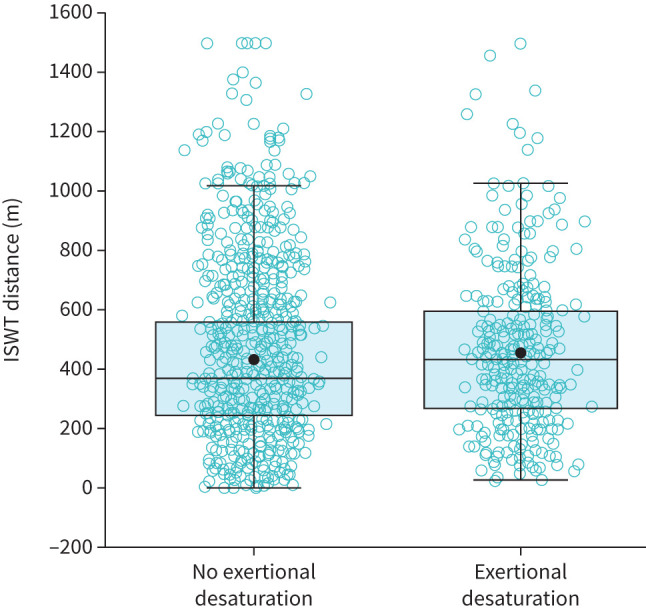
Incremental shuttle walk test (ISWT) distance in those with and without exertional desaturation. Open circles represent individual data; closed circles represent mean values. Boxes represent median and interquartile range (IQR). Whiskers represent range within 1.5 IQR.

At 5 months post-discharge, a pre- to post-test drop in *S*_pO_2__ of ≥4% was observed in 333/1470 (22.7%) participants, with 102/1470 (6.9%) participants experiencing this reduction in *S*_pO_2__ with a *S*_pO_2__ <90%. A further 3/1470 (0.2%) patients finished the test with a *S*_pO_2__ <90% without a ≥4% reduction in *S*_pO_2__; 8/1470 (0.5%) tests were terminated due to a *S*_pO_2__ ≤80%. Thus, in total, exertional desaturation was observed in 336/1470 (22.9%) participants. The number of adults with a pre-existing respiratory comorbidity (*e.g.* COPD, asthma, interstitial lung disease, bronchiectasis, obstructive sleep apnoea, obesity hyperventilation syndrome or pleural effusion) was not different between those with and without exertional desaturation (exertional desaturation: 94/336 (28.0%) patients had a respiratory comorbidity; no exertional desaturation: 287/1134 (25.3%) patients had a respiratory comorbidity; p=0.33). ISWT distance measured 5 months post-discharge was not different between individuals with and without exertional desaturation (p=0.17) (figure 1).

At a separate research visit 12 months post-discharge (median (IQR) 12.4 (11.7–13.2) months), 904/1470 (61.5%) participants performed an ISWT with *S*_pO_2__ measured pre- and post-test. There was no difference in the prevalence of exertional desaturation in this cohort at 5 months (209/904 (23.1%)) participants) and 12 months (191/904 (21.1%) participants) post-discharge (p=0.23).

### Repeatability of the ISWT

At 5 months post-discharge, 1034/1593 (64.9%) participants performed a second ISWT. Mean±sd distance walked was greater in the second ISWT (451±260 m) compared to the first ISWT (425±243 m; p=0.01), with a mean±sd change of 27±97 m. On the participants’ second ISWT, 58.1% (n=601) increased their distance by ≥10 m, 30.9% (n=319) reduced their distance by ≥10 m and 11.0% (n=114) maintained their distance. Resting heart rate and self-reported leg fatigue were greater before the second ISWT compared to the first test (median (IQR) resting heart rate: ISWT1: 74 (66–84) beats·min^−1^; ISWT2: 79 (70–89) beats·min^−1^; median (IQR) Borg leg fatigue: ISWT1: 0 (0–1); ISWT2: 0 (0–1); p=0.01). Borg breathlessness was not different before both tests (median (IQR): ISWT1: 0 (0–1); ISWT2: 0.5 (0–1); p=0.29).

When a linear regression on the absolute difference between the first and second ISWT was performed (R^2^=0.03), heteroscedasticity was present (p=0.01). The coefficient of repeatability was 268 m. Test–retest variability was 15.6%, with an ICC estimate of 0.96 (95% CI 0.95–0.97).

**FIGURE 2:**
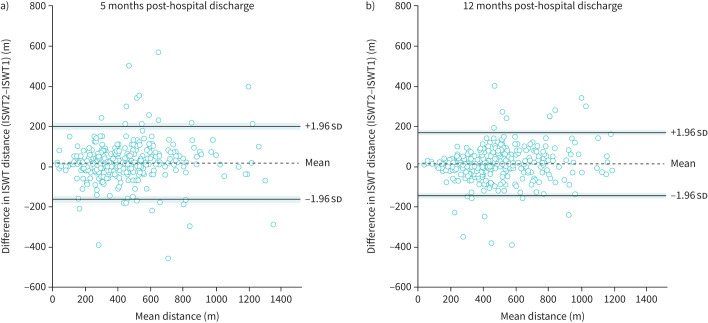
Repeatability of the incremental shuttle walk test (ISWT) at a) 5 and b) 12 months post-hospital discharge for COVID-19. Black dashed line represents mean change in ISWT distance between ISWT2 and ISWT1. Black solid lines denote upper and lower (95% CI) limits with shading representing the precision of these limits (95% CI of limits).

Of the 1034 participants who performed two ISWTs at 5 months post-discharge, 390 (37.7%) participants performed two ISWTs at 12 months post-discharge. ISWT distance increased between ISWT1 and ISWT2 at 12 months post-discharge, with no difference in the magnitude of increase from the 5-month visit (mean±sd difference between ISWT2 and ISWT1: 5 months: 19±94 m; 12 months: 11±80 m; time effect: p=0.01; interaction: p=0.30). The Bland–Altman plots (figure 2) show that the limits of agreement for these mean differences were −165–202 and −146–168 m at 5 and 12 months, respectively. Heteroscedasticity was present at both time-points (p<0.05) (figure 2). The repeatability coefficient was 259 and 222 m at 5 and 12 months post-discharge, respectively. Test–retest variability was 15.5% at 5 months and 12.3% at 12 months post-discharge, with an ICC estimate of 0.96 (95% CI 0.95–0.97) at 5 months and 0.97 (95% CI 0.96–0.97) at 12 months post-hospital discharge.

## Discussion

This is the first study to explore the safety, physiological response and repeatability of the ISWT in individuals previously hospitalised with COVID-19. The results suggest that the ISWT is safe in this cohort, with expected exercise-induced changes in physiological variables observed and an extremely low number of adverse events reported. The results also demonstrate an increase in ISWT distance between the first and second test performed at both 5 and 12 months post-discharge, suggesting a learning effect is present.

Patient-reported reasons for intolerance support test safety in this cohort. More than half of the participants (826 (51.8%)) were limited, at least in part, due to breathlessness, which is one of the most prevalent symptoms reported in COVID-19 survivors [7]. After breathlessness, the most common reasons contributing to test termination were walking speed, leg fatigue and musculoskeletal pain, which are often reported at test termination in respiratory diseases [29]. In line with almost a third of participants being limited by leg fatigue, data from cardiopulmonary exercise tests suggest that muscle deconditioning and peripheral factors may contribute to intolerance in this cohort [30, 31].

In terms of the cardiovascular response to the ISWT, heart rate and systolic blood pressure increased pre- to post-exercise within expected limits [32–34]. Heart rate at intolerance was lower than the predicted maximum values calculated using the participants’ age. Subjective ratings of leg fatigue and breathlessness also increased pre- to post-exercise but were “moderate” at test termination. Greater degrees of breathlessness have been seen immediately post-test in individuals with COPD and interstitial lung disease [14, 35], but not in those with bronchiectasis [36]. The ISWT is usually described as a maximal test, with no difference between peak oxygen uptake measured in the ISWT compared to a cardiopulmonary exercise test in those with respiratory conditions [14, 37, 38]. However, heart rate, leg fatigue and breathlessness measured post-test in this study indicate a submaximal test. Reasons underpinning this submaximal performance may include concern of post-exertional symptom exacerbation, low exercise confidence, general fatigue, lack of test familiarisation or reduced motivation to exercise. Future work should compare ISWT and cardiopulmonary exercise test responses to assess ISWT validity in COVID-19 survivors.

Overall for the population, peripheral oxygen saturation only reduced by a median of 1% pre- to post-test at 5 months post-discharge; however, nearly one in four (22.9%) participants experienced exertional desaturation. In another cohort study, after a 6MWT test, rather than an ISWT, a ≥4% decrease in *S*_pO_2__ was seen in 6/27 (22%) patients 5.8 months post-hospital discharge for COVID-19 [39]. Higher instances of this magnitude of exertional desaturation have been reported at hospital discharge and 1 month post-discharge, with 43% and 32% of patients affected, respectively [40, 41]. These findings suggest that the prevalence of exertional desaturation when defined as a ≥4% fall in *S*_pO_2__ may decrease in COVID-19 survivors during the first 5 months of recovery. The 7% of participants with an *S*_pO_2__ >90% after the ISWT at 5 months post-discharge mirrors the 7% of previously hospitalised COVID-19 survivors with an *S*_pO_2__ ≤88% after a 6MWT at 12 weeks post-symptom onset [42]. The lack of change in the prevalence of exertional desaturation between 5 and 12 months post-discharge in this cohort aligns with the prevalence of their self-reported symptoms remaining largely unchanged during this period [5].

The distribution of pre-existing respiratory comorbidities, between individuals with and without exertional desaturation, was similar in this study, as total number of comorbidities has been reported previously [40]. The mechanisms underpinning exertional desaturation in individuals without respiratory disease before their COVID-19 infection remain unknown. However, the respiratory sequelae of COVID-19, such as interstitial lung abnormalities, which reduce diffusion capacity, may contribute [6, 43]. ISWT distance was not different between those with and without exertional desaturation, suggesting exertional desaturation does not play a dominant role in determining exercise intolerance in this patient population.

ISWT distance is repeatable after a practice test in several clinical populations, including COPD, chronic heart failure and obstructive sleep apnoea [13, 15, 17, 22, 44]. The mean increase in distance achieved in the second ISWT compared to the first test was 27 and 11 m at 5 and 12 months post-discharge, and therefore may not influence exercise prescription. However, the Bland–Altman plots (figure 2) have wide limits of agreement, suggesting some large differences in the distance achieved between tests were present. These differences were bidirectional, with 30% of participants achieving a shorter distance in the second test at 5 months post-discharge. In line with this observation, 25/51 (49%) individuals with long COVID who were hospitalised during their acute infection achieved a shorter distance in their second 6MWT compared to their first test when test repeatability was assessed [45]. Although resting heart rate and patient-reported leg fatigue were statistically greater before the second test compared to the first test at 5 months post-discharge in the current study, these differences were not physiologically significant (median (IQR) Borg leg fatigue values were 0 (0–1) for both tests), thus ruling out inadequate recovery as a reason for reduced performance. It is plausible that reduced motivation or fear of symptom exacerbation reduced performance in the second test.

As an increase in distance was observed after the first test with large differences between distances present, a familiarisation test is recommended to ensure a true measurement of the ability to tolerate the test is recorded. In turn, this will afford an appropriate exercise prescription to be given and may increase the sensitivity of the ISWT to discriminate a pre- to post-intervention change in exercise tolerance. Whilst one familiarisation ISWT is recommended in several clinical populations [13, 15, 22], without assessing the repeatability between the second ISWT and an additional third test, the number of familiarisation tests required in the post-COVID-19 cohort remains unclear. The high coefficient of repeatability and presence of heteroscedasticity also need to be considered when interpreting whether a meaningful change has occurred in ISWT distance pre- to post-intervention.

### Limitations

At the 5-month visit, 559 (35.1%) participants did not perform a second ISWT for reasons unrecorded, potentially reducing the generalisability of the results. This study was in individuals previously hospitalised due to COVID-19, therefore the data on ISWT safety may not directly relate to non-hospitalised cohorts. In the absence of operator proficiency tests, we cannot rule out that the pressures of the pandemic and rapid set-up of the study resulted in suboptimal operator training affecting reliability. However, all study sites received a training video and standard operating procedure, with test instructions provided to the participant by an audio tape, to ensure that the test was consistently employed by researchers in accordance with technical standards [19, 22]. Resting heart rate and Borg leg fatigue were statistically greater before ISWT1 compared to ISWT2 at 5 months post-discharge. Although these differences were unlikely physiologically significant, their presence compromises otherwise identical test conditions. Finally, post-exertional symptom exacerbation was not assessed, negating any insight into whether the ISWT effects post-exertional symptom exacerbation.

### Conclusions

The ISWT appears to be safe in individuals following a COVID-19 hospitalisation, providing support for its use in COVID-19 assessment and rehabilitation settings. An increase in ISWT distance with a high repeatability coefficient was apparent between the first and second ISWT at 5 and 12 months post-discharge, therefore a familiarisation walk is recommended in this cohort. Future research should focus on assessing the number of familiarisation tests needed to achieve within-day repeatability.

## Supplementary material

**Please note:** supplementary material is not edited by the Editorial Office, and is uploaded as it has been supplied by the author. PHOSP-COVID Collaborative Group 00089-2025.SUPPLEMENT Collaborators 00089-2025.SUPPLEMENT2
