# Supplementary material for: The safety, physiological response and repeatability of the incremental shuttle walk test in survivors of COVID-19
Source: ERJ Open Res. 2025 Dec 15;11(6):00089-2025. doi: 10.1183/23120541.00089-2025 (PMC12704175; doi:10.1183/23120541.00089-2025)
Supplement: Supplementary file 2 [file 00089-2025.SUPPLEMENT2.pdf]

Affiliated Organisation	Initial + Name	Initial	Forename	Surname
(1) Usher Institute, University of Edinburgh, Edinburgh, United Kingdom and (2) Royal Infirmary of Edinburgh, NHS Lothian, Edinburgh, United Kingdom	N I Lone	N I	Nazir	Lone
1. Baillie Gifford Pandemic Science Hub, Centre for Inflammation Research, The Queen's Medical Research Institute, University of Edinburgh, Edinburgh, UK<br>2. MRC Human Genetics Unit, Institute of Genetics and Cancer, University of Edinburgh, Western General Hospital, Edinburgh, UK<br>3. Roslin Institute, University of Edinburgh, Edinburgh, UK<br>4. Intensive Care Unit, Royal Infirmary of Edinburgh, Edinburgh, UK	J K Baillie	J K	Kenneth	Baillie
1. Baillie Gifford Pandemic Science Hub, Centre for Inflammation Research, The Queen's Medical Research Institute, University of Edinburgh, Edinburgh, UK<br>2. Roslin Institute, University of Edinburgh, Edinburgh, UK	E Pairo-Castineira	E P-C	Erola	Pairo-Castineira
1. Baillie Gifford Pandemic Science Hub, Centre for Inflammation Research, The Queen's Medical Research Institute, University of Edinburgh, Edinburgh, UK<br>2. Roslin Institute, University of Edinburgh, Edinburgh, UK	N Avramidis	N-A	Nikos	Avramidis
1. Department of Population Health Sciences, University of Leicester, Leicester, UK<br>2. NIHR Leicester Biomedical Research Centre, Leicester, UK	L V Wain	L V	Louise	Wain
1. Department of Population Health Sciences, University of Leicester, Leicester, UK<br>2. NIHR Leicester Biomedical Research Centre, Leicester, UK	B Guillen-Guio	B	Beatriz	Guillen-Guio
1. Department of Population Health Sciences, University of Leicester, Leicester, UK<br>2. NIHR Leicester Biomedical Research Centre, Leicester, UK	O C Leavy	O C	Olivia	Leavy
Action for Pulmonary Fibrosis	S Jones	S		Jones
Airedale NHS Foundation Trust	L Armstrong	L	Lisa	Armstrong
Airedale NHS Foundation Trust	B Hairsine	B	Brigid	Hairsine
Airedale NHS Foundation Trust	H Henson	H	Helen	Henson
Airedale NHS Foundation Trust	C Kurasz	C	Claire	Kurasz
Airedale NHS Foundation Trust	A Shaw	A	Alison	Shaw

Airedale NHS Foundation Trust	L Shenton	L	Liz	Shenton
Alzheimer's Research UK	H Dobson	H	Hannah	Dobson
Aneurin Bevan University Health Board	A Dell	A	Amanda	Dell
Aneurin Bevan University Health Board	S Fairbairn	S	Sara	Fairbairn
Aneurin Bevan University Health Board	N Hawkings	N	Nancy	Hawkings
Aneurin Bevan University Health Board	J Haworth	J	Jill	Haworth
Aneurin Bevan University Health Board	M Hoare	M	Michaela	Hoare
Aneurin Bevan University Health Board	V Lewis	V	Victoria	Lewis
Aneurin Bevan University Health Board	A Lucey	A	Alice	Lucey
Aneurin Bevan University Health Board	G Mallison	G	Georgia	Mallison
Aneurin Bevan University Health Board	H Nassa	H	Heeah	Nassa
Aneurin Bevan University Health Board	C Pennington	C	Chris	Pennington
Aneurin Bevan University Health Board	A Price	A	Andrea	Price
Aneurin Bevan University Health Board	C Price	C	Claire	Price
Aneurin Bevan University Health Board	A Storrie	A	Andrew	Storrie
Aneurin Bevan University Health Board	G Willis	G	Gemma	Willis
Aneurin Bevan University Health Board	S Young	S	Susan	Young
Asthma UK and British Lung Foundation Partnership	K Poinasamy	K	Krisnah	Poinasamy
Asthma UK and British Lung Foundation Partnership	S Walker	S	Samantha	Walker
Asthma UK BLF	I Jarrold	I	Ian	Jarrold
Baillie Gifford Pandemic Science Hub, Centre for Inflammation Research, The Queen’s Medical Research Institute, University of Edinburgh, Edinburgh, UK	K Rawlik	K-R	Konrad	Rawlik
Barnsley Hospital NHS Foundation Trust	A Sanderson	A	Amy	Sanderson
Barts Health NHS Trust	K Chong-James	K		Chong-James
Barts Health NHS Trust	C David	C		David
Barts Health NHS Trust	W Y James	W Y		James
Barts Health NHS Trust	P Pfeffer	P	Paul	Pfeffer
Barts Health NHS Trust	O Zongo	O		Zongo
Barts Health NHS Trust & Queen Mary University of London	A Martineau	A	Adrian	Martineau

	Barts Heart Centre	C Manisty	C		Manisty
	Belfast Health & Social Care Trust & Queen's University Belfast	C Armour	C	Cherie	Armour
	Belfast Health & Social Care Trust & Queen's University Belfast	V Brown	V	Vanessa	Brown
	Belfast Health & Social Care Trust & Queen's University Belfast	J Busby	J	John	Busby
	Belfast Health & Social Care Trust & Queen's University Belfast	B Connolly	B	Bronwen	Connolly
	Belfast Health & Social Care Trust & Queen's University Belfast	T Craig	T	Thelma	Craig
	Belfast Health & Social Care Trust & Queen's University Belfast	S Drain	S	Stephen	Drain
	Belfast Health & Social Care Trust & Queen's University Belfast	L G Heaney	L G	Liam	Heaney
	Belfast Health & Social Care Trust & Queen's University Belfast	B King	B	Bernie	King
	Belfast Health & Social Care Trust & Queen's University Belfast	N Magee	N	Nick	Magee
	Belfast Health & Social Care Trust & Queen's University Belfast	E Major	E		Major
	Belfast Health & Social Care Trust & Queen's University Belfast	D McAulay	D	Danny	McAulay
	Belfast Health & Social Care Trust & Queen's University Belfast	L Mcgarvey	L	Lorcan	McGarvey
	Belfast Health & Social Care Trust & Queen's University Belfast	J McGinness	J	Jade	McGinness
	Belfast Health & Social Care Trust & Queen's University Belfast	T Peto	T	Tunde	Peto
	Belfast Health & Social Care Trust & Queen's University Belfast	R Stone	R	Roisin	Stone
	Betsi Cadwallader University Health Board	A Bolger	A	Annette	Bolger
	Betsi Cadwallader University Health Board	F Davies	F	Ffyon	Davies
	Betsi Cadwallader University Health Board	A Haggar	A	Ahmed	Haggar
	Betsi Cadwallader University Health Board	J Lewis	J	Joanne	Lewis
	Betsi Cadwallader University Health Board	A Lloyd	A	Arwel	Lloyd
	Betsi Cadwallader University Health Board	R Manley	R		Manley
	Betsi Cadwallader University Health Board	E McIvor	E	Emma	McIvor
	Betsi Cadwallader University Health Board	D Menzies	D	Daniel	Menzies
	Betsi Cadwallader University Health Board	K Roberts	K		Roberts
	Betsi Cadwallader University Health Board	W Saxon	W		Saxon
	Betsi Cadwallader University Health Board	D Southern	D	David	Southern
	Betsi Cadwallader University Health Board	C Subbe	C	Christian	Subbe
	Betsi Cadwallader University Health Board	V Whitehead	V	Victoria	Whitehead

BHF Centre for Cardiovascular Science, University of Edinburgh	A Bularga	A	Anda	Bularga
BHF Centre for Cardiovascular Science, Usher Institute of Population Health Sciences and Informatics, University of Edinburgh	N L Mills	N L	Nicholas	Mills
Borders General Hospital, NHS Borders	J Dawson	J	Joy	Dawson
Borders General Hospital, NHS Borders	H El-Taweel	H	Hosni	El-Taweel
Borders General Hospital, NHS Borders	L Robinson	L	Leanne	Robinson
Bradford Teaching Hospitals NHS Foundation Trust	L Brear	L	Lucy	Brear
Bradford Teaching Hospitals NHS Foundation Trust	K Regan	K	Karen	Regan
Bradford Teaching Hospitals NHS Foundation Trust	D Saralaya	D	Dinesh	Saralaya
Bradford Teaching Hospitals NHS Foundation Trust	K Storton	K	Kim	Storton
British Heart Foundation	S Amoils	S	Shannon	Amoils
Cambridge University Hospitals NHS Foundation Trust, NIHR Cambridge Clinical Research Facility & University of Cambridge	A Bermperi	A	Areti	Bermperi
Cambridge University Hospitals NHS Foundation Trust, NIHR Cambridge Clinical Research Facility & University of Cambridge	I Cruz	I	Isabel	Cruz
Cambridge University Hospitals NHS Foundation Trust, NIHR Cambridge Clinical Research Facility & University of Cambridge	K Dempsey	K		Dempsey
Cambridge University Hospitals NHS Foundation Trust, NIHR Cambridge Clinical Research Facility & University of Cambridge	A Elmer	A	Anne	Elmer
Cambridge University Hospitals NHS Foundation Trust, NIHR Cambridge Clinical Research Facility & University of Cambridge	J Fuld	J	Jonathon	Fuld
Cambridge University Hospitals NHS Foundation Trust, NIHR Cambridge Clinical Research Facility & University of Cambridge	H Jones	H		Jones
Cambridge University Hospitals NHS Foundation Trust, NIHR Cambridge Clinical Research Facility & University of Cambridge	S Jose	S	Sherly	Jose
Cambridge University Hospitals NHS Foundation Trust, NIHR Cambridge Clinical Research Facility & University of Cambridge	S Marciniak	S	Stefan	Marciniak
Cambridge University Hospitals NHS Foundation Trust, NIHR Cambridge Clinical Research Facility & University of Cambridge	M Parkes	M		Parkes
Cambridge University Hospitals NHS Foundation Trust, NIHR Cambridge Clinical Research Facility & University of Cambridge	C Ribeiro	C	Carla	Ribeiro

	Cambridge University Hospitals NHS Foundation Trust, NIHR Cambridge Clinical Research Facility & University of Cambridge	J Taylor	J	Jessica	Taylor
	Cambridge University Hospitals NHS Foundation Trust, NIHR Cambridge Clinical Research Facility & University of Cambridge	M Toshner	M	Mark	Toshner
	Cambridge University Hospitals NHS Foundation Trust, NIHR Cambridge Clinical Research Facility & University of Cambridge	L Watson	L		Watson
	Cambridge University Hospitals NHS Foundation Trust, NIHR Cambridge Clinical Research Facility & University of Cambridge	J Worsley	J		Worsley
	Cardiff and Vale University Health Board	L Broad	L	Lauren	Broad
	Cardiff and Vale University Health Board	T Evans	T	Teriann	Evans
	Cardiff and Vale University Health Board	M Haynes	M	Matthew	Haynes
	Cardiff and Vale University Health Board	L Jones	L		Jones
	Cardiff and Vale University Health Board	L Knibbs	L	Lucy	Knibbs
	Cardiff and Vale University Health Board	A McQueen	A	Alison	McQueen
	Cardiff and Vale University Health Board	C Oliver	C	Catherine	Oliver
	Cardiff and Vale University Health Board	K Paradowski	K	Kerry	Paradowski
	Cardiff and Vale University Health Board	R Sabit	R	Ramsey	Sabit
	Cardiff and Vale University Health Board	J Williams	J	Jenny	Williams
	Cardiff University, National Centre for Mental Health	I Jones	I	Ian	Jones
	CEO at MQ Mental Health Research,	L Milligan	L	Lea	Milligan
	Chesterfield Royal Hospital NHS Trust	E Harris	E	Edward	Harris
	Chesterfield Royal Hospital NHS Trust	C Sampson	C	Claire	Sampson
	Cwm Taf Morgannwg University Health Board	E Davies	E	Ellie	Davies
	Cwm Taf Morgannwg University Health Board	C Evenden	C	Cerys	Evenden
	Cwm Taf Morgannwg University Health Board	A Hancock	A	Alyson	Hancock
	Cwm Taf Morgannwg University Health Board	K Hancock	K	Kia	Hancock
	Cwm Taf Morgannwg University Health Board	C Lynch	C	Ceri	Lynch
	Cwm Taf Morgannwg University Health Board	M Rees	M	Meryl	Rees
	Cwm Taf Morgannwg University Health Board	L Roche	L	Lisa	Roche
	Cwm Taf Morgannwg University Health Board	N Stroud	N	Natalie	Stroud

Cwm Taf Morgannwg University Health Board	T Thomas-Woods	T		Thomas-Woods
Department of Oncology and Metabolism, University of Sheffield, Sheffield, UK	S Heller	S	Simon	Heller
Department of Psychological Medicine, Institute of Psychiatry, Psychology and Neuroscience, King's College London, London, United Kingdom	T Chalder	T	Trudie	Chalder
Diabetes UK	K Shah	K	Kamini	Shah
Diabetes UK, University of Glasgow	E Robertson	E	Elizabeth	Robertson
DUK | NHS Digital, Salford Royal Foundation Trust	B Young	B	Bob	Young
East Cheshire NHS Trust	M Babores	M	Marta	Babores
East Cheshire NHS Trust	M Holland	M	Maureen	Holland
East Cheshire NHS Trust	N Keenan	N	Natalie	Keenan
East Cheshire NHS Trust	S Shashaa	S	Sharlene	Shashaa
East Cheshire NHS Trust	H Wassall	H	Helen	Wassall
East Kent Hospitals University NHS Foundation Trust	L Austin	L	Liam	Austin
East Kent Hospitals University NHS Foundation Trust	E Beranova	E	Eva	Beranova
East Kent Hospitals University NHS Foundation Trust	T Cosier	T	Tracey	Cosier
East Kent Hospitals University NHS Foundation Trust	J Deery	J	Joanne	Deery
East Kent Hospitals University NHS Foundation Trust	T Hazelton	T	Tracy	Hazelton
East Kent Hospitals University NHS Foundation Trust	C Price	C	Carly	Price
East Kent Hospitals University NHS Foundation Trust	H Ramos	H	Hazel	Ramos
East Kent Hospitals University NHS Foundation Trust	R Solly	R	Reanne	Solly
East Kent Hospitals University NHS Foundation Trust	S Turney	S	Sharon	Turney
East Kent Hospitals University NHS Foundation Trust	H Weston	H	Heather	Weston
Faculty of Medicine, Nursing and Health Sciences, School of Biomedical Sciences, Monash University, Melbourne, Australia	E Coughlan	E-C	Eamon	Coughlan
Francis Crick Institute	M Ralser	M	Markus	Ralser
Gateshead NHS Trust	L Pearce	L	Lorraine	Pearce
Gateshead NHS Trust	S Pugmire	S		Pugmire
Gateshead NHS Trust	W Stoker	W	Wendy	Stoker

Gateshead NHS Trust	A Wilson	A	Ann	Wilson
Gateshead NHS Trust	W McCormick	W		McCormick
Great Western Hospital Foundation Trust	E Fraile	E	Eva	Fraile
Great Western Hospital Foundation Trust	J Ugoji	J	Jacinta	Ugoji
Guy's and St Thomas' NHS Foundation Trust	L AguilarJimenez	L A	Laura	Aguilar Jimenez
Guy's and St Thomas' NHS Foundation Trust	G Arbane	G	Gill	Arbane
Guy's and St Thomas' NHS Foundation Trust	S Betts	S	Sarah	Betts
Guy's and St Thomas' NHS Foundation Trust	K Bisnauthsing	K	Karen	Bisnauthsing
Guy's and St Thomas' NHS Foundation Trust	A Dewar	A		Dewar
Guy's and St Thomas' NHS Foundation Trust	N Hart	N	Nicholas	Hart
Guy's and St Thomas' NHS Foundation Trust	G Kaltsakas	G		Kaltsakas
Guy's and St Thomas' NHS Foundation Trust	H Kerslake	H	Helen	Kerslake
Guy's and St Thomas' NHS Foundation Trust	MM Magtoto	M M	Murphy	Magtoto
Guy's and St Thomas' NHS Foundation Trust	P Marino	P	Philip	Marino
Guy's and St Thomas' NHS Foundation Trust	LM Martinez	L M		Martinez
Guy's and St Thomas' NHS Foundation Trust	M Ostermann	M	Marlies	Ostermann
Guy's and St Thomas' NHS Foundation Trust	J Rossdale	J	Jennifer	Rossdale
Guy's and St Thomas' NHS Foundation Trust	TS Solano	T S	Teresa	Solano
Hampshire Hospitals NHS Foundation Trust	M Alvarez Corral	M	Maria	Alvarez Corral
Hampshire Hospitals NHS Foundation Trust	A Arias	A	Ava Maria	Arias
Hampshire Hospitals NHS Foundation Trust	E Bevan	E	Emily	Bevan
Hampshire Hospitals NHS Foundation Trust	D Griffin	D	Denise	Griffin
Hampshire Hospitals NHS Foundation Trust	J Martin	J	Jane	Martin
Hampshire Hospitals NHS Foundation Trust	J Owen	J		Owen
Hampshire Hospitals NHS Foundation Trust	S Payne	S	Sheila	Payne
Hampshire Hospitals NHS Foundation Trust	A Prabhu	A		Prabhu

	Hampshire Hospitals NHS Foundation Trust	A Reed	A	Annabel	Reed
	Hampshire Hospitals NHS Foundation Trust	W Storrar	W	Will	Storrar
	Hampshire Hospitals NHS Foundation Trust	N Williams	N	Nick	Williams
	Hampshire Hospitals NHS Foundation Trust	C Wrey Brown	C	Caroline	Wrey Brown
	Harrogate and District NHD Foundation Trust	T Burdett	T	Tracy	Burdett
	Harrogate and District NHD Foundation Trust	J Featherstone	J	James	Featherstone
	Harrogate and District NHD Foundation Trust	C Lawson	C	Cathy	Lawson
	Harrogate and District NHD Foundation Trust	A Layton	A	Alison	Layton
	Harrogate and District NHD Foundation Trust	C Mills	C	Clare	Mills
	Harrogate and District NHD Foundation Trust	L Stephenson	L	Lorraine	Stephenson
	Health & Care Research Wales	Y Ellis	Y	Yvette	Ellis
	Hull University Teaching Hospitals NHS Trust & University of Hull	P Atkin	P	Paul	Atkin
	Hull University Teaching Hospitals NHS Trust & University of Hull	K Brindle	K		Brindle
	Hull University Teaching Hospitals NHS Trust & University of Hull	M G Crook	M G	Michael	Crooks
	Hull University Teaching Hospitals NHS Trust & University of Hull	K Drury	K	Katie	Drury
	Hull University Teaching Hospitals NHS Trust & University of Hull	N Easom	N	Nicholas	Easom
	Hull University Teaching Hospitals NHS Trust & University of Hull	R Flockton	R	Rachel	Flockton
	Hull University Teaching Hospitals NHS Trust & University of Hull	L Holdsworth	L		Holdsworth
	Hull University Teaching Hospitals NHS Trust & University of Hull	A Richards	A		Richards
	Hull University Teaching Hospitals NHS Trust & University of Hull	D L Sykes	D L		Sykes
	Hull University Teaching Hospitals NHS Trust & University of Hull	S Thackray-Nocera	S	Susannah	Thackray-Nocera
	Hull University Teaching Hospitals NHS Trust & University of Hull	C Wright	C		Wright
	Hywel Dda University Health Board	S Coetzee	S		Coetzee
	Hywel Dda University Health Board	K Davies	K	Kim	Davies
	Hywel Dda University Health Board	R Hughes	R A	Rachel Ann	Hughes
	Hywel Dda University Health Board	R Loosley	R	Ronda	Loosley

	Hywel Dda University Health Board	H McGuinness	H	Heather	McGuinness
	Hywel Dda University Health Board	A Mohamed	A	Abdelrahman	Mohamed
	Hywel Dda University Health Board	L O'Brien	L	Linda	O'Brien
	Hywel Dda University Health Board	Z Omar	Z	Zohra	Omar
	Hywel Dda University Health Board	E Perkins	E	Emma	Perkins
	Hywel Dda University Health Board	J Phipps	J	Janet	Phipps
	Hywel Dda University Health Board	G Ross	G	Gavin	Ross
	Hywel Dda University Health Board	A Taylor	A	Abigail	Taylor
	Hywel Dda University Health Board	H Tench	H	Helen	Tench
	Hywel Dda University Health Board	R Wolf-Roberts	R	Rebecca	Wolf-Roberts
	Imperial College Healthcare NHS Trust & Imperial College London	L Burden	L		Burden
	Imperial College Healthcare NHS Trust & Imperial College London	E Calvelo	E	Ellen	Calvelo
	Imperial College Healthcare NHS Trust & Imperial College London	B Card	B	Bethany	Card
	Imperial College Healthcare NHS Trust & Imperial College London	C Carr	C	Caitlin	Carr
	Imperial College Healthcare NHS Trust & Imperial College London	E R Chilvers	E R	Edwin	Chilvers
	Imperial College Healthcare NHS Trust & Imperial College London	D Copeland	D	Donna	Copeland
	Imperial College Healthcare NHS Trust & Imperial College London	P Cullinan	P		Cullinan
	Imperial College Healthcare NHS Trust & Imperial College London	P Daly	P	Patrick	Daly
	Imperial College Healthcare NHS Trust & Imperial College London	L Evison	L	Lynsey	Evison
	Imperial College Healthcare NHS Trust & Imperial College London	T Fayzan	T	Tamanah	Fayzan
	Imperial College Healthcare NHS Trust & Imperial College London	H Gordon	H	Hussain	Gordon
	Imperial College Healthcare NHS Trust & Imperial College London	S Haq	S	Sulaimaan	Haq
	Imperial College Healthcare NHS Trust & Imperial College London	R G Jenkins	R G	Gisli	Jenkins
	Imperial College Healthcare NHS Trust & Imperial College London	C King	C	Clara	King
	Imperial College Healthcare NHS Trust & Imperial College London	O Kon	O	Onn Min	Kon
	Imperial College Healthcare NHS Trust & Imperial College London	K March	K	Katherine	March

	Imperial College Healthcare NHS Trust & Imperial College London	M Mariveles	M	Myril	Mariveles
	Imperial College Healthcare NHS Trust & Imperial College London	L McLeavey	L	Laura	McLeavey
	Imperial College Healthcare NHS Trust & Imperial College London	N Mohamed	N	Noura	Mohamed
	Imperial College Healthcare NHS Trust & Imperial College London	S Moriera	S	Silvia	Moriera
	Imperial College Healthcare NHS Trust & Imperial College London	U Munawar	U	Unber	Munawar
	Imperial College Healthcare NHS Trust & Imperial College London	J Nunag	J L	Jose Lloyd	Nunag
	Imperial College Healthcare NHS Trust & Imperial College London	U Nwanguma	U	Uchechi	Nwanguma
	Imperial College Healthcare NHS Trust & Imperial College London	L Orriss-Dib	L	Lorna	Orriss-Dib
	Imperial College Healthcare NHS Trust & Imperial College London	A Ross	A	Alexandra	Ross
	Imperial College Healthcare NHS Trust & Imperial College London	M Roy	M	Maura	Roy
	Imperial College Healthcare NHS Trust & Imperial College London	E Russell	E	Emily	Russell
	Imperial College Healthcare NHS Trust & Imperial College London	K Samuel	K	Katherine	Samuel
	Imperial College Healthcare NHS Trust & Imperial College London	J Schronce	J		Schronce
	Imperial College Healthcare NHS Trust & Imperial College London	N Simpson	N	Neil	Simpson
	Imperial College Healthcare NHS Trust & Imperial College London	L Tarusan	L	Lawrence	Tarusan
	Imperial College Healthcare NHS Trust & Imperial College London	D C Thomas	D C	David	Thomas
	Imperial College Healthcare NHS Trust & Imperial College London	C Wood	C	Chloe	Wood
	Imperial College Healthcare NHS Trust & Imperial College London	N Yasmin	N	Najira	Yasmin
	Imperial College London	D Altmann	D	Danny	Altmann
	Imperial College London	L S Howard	L S	Luke	Howard
	Imperial College London	D Johnston	D	Desmond	Johnston
	Imperial College London	A Lingford-Hughes	A	Anne	Lingford-Hughes
	Imperial College London	W D-C Man	W D-C	William	Man
	Imperial College London	J Mitchell	J	Jane	Mitchell
	Imperial College London	P L Molyneaux	P L	Philip	Molyneaux
	Imperial College London	C Nicolaou	C	Christos	Nicolaou
	Imperial College London	D P O'Regan	D P		O'Regan

Imperial College London	L Price	L		Price
Imperial College London	J Quint	J	Jenni	Quint
Imperial College London	D Smith	D	David	Smith
Imperial College London	R S Thwaites	R S	Ryan	Thwaites
Imperial College London	J Valabhji	J	Jonathon	Valabhji
Imperial College London	S Walsh	S	Simon	Walsh
Imperial College London	C M Efstathiou	C M	Claudia	Efstathiou
Imperial College London	F Liew	F	Felicity	Liew
Imperial College London	A Frankel	A	Anew	Frankel
Imperial College London	L Lightstone	L	Liz	Lightstone
Imperial College London	S McAdoo	S	Steve	McAdoo
Imperial College London	M Wilkins	M	Martin	Wilkins
Imperial College London	M Willicombe	M	Michelle	Willicombe
Institute of Cardiovascular & Medical Sciences, BHF Glasgow Cardiovoascular Research Centre, Universitty of Glasgow	R Touyz	R		Touyz
Kettering General Hospital NHS Trust	A-M Guerdette	A-M	Anne-Marie	Guerdette
Kettering General Hospital NHS Trust	M Hewitt	M	Melanie	Hewitt
Kettering General Hospital NHS Trust	R Reddy	R		Reddy
Kettering General Hospital NHS Trust	K Warwick	K	Katie	Warwick
Kettering General Hospital NHS Trust	S White	S	Sonia	White
Kidney Research UK	A McMahon	A	Aisling	McMahon
King’s College Hospital NHS Foundation Trust & Kings College London	O Adeyemi	O	Oluwaseun	Adeyemi
King’s College Hospital NHS Foundation Trust & Kings College London	R Adrego	R	Rita	Adrego
King’s College Hospital NHS Foundation Trust & Kings College London	H Assefa-Kebede	H	Hosanna	Assefa-Kebede
King’s College Hospital NHS Foundation Trust & Kings College London	J Breeze	J	Jonathon	Breeze
King’s College Hospital NHS Foundation Trust & Kings College London	S Byrne	S		Byrne
King’s College Hospital NHS Foundation Trust & Kings College London	P Dulawan	P	Pearl	Dulawan

King’s College Hospital NHS Foundation Trust & Kings College London	A Hoare	A	Amy	Hoare
King’s College Hospital NHS Foundation Trust & Kings College London	C J Jolley	C J	Caroline	Jolley
King’s College Hospital NHS Foundation Trust & Kings College London	A Knighton	A	Abigail	Knighton
King’s College Hospital NHS Foundation Trust & Kings College London	S Patale	S	Sheetal	Patale
King’s College Hospital NHS Foundation Trust & Kings College London	I Peralta	I	Ida	Peralta
King’s College Hospital NHS Foundation Trust & Kings College London	N Powell	N	Natassia	Powell
King’s College Hospital NHS Foundation Trust & Kings College London	A Ramos	A	Albert	Ramos
King’s College Hospital NHS Foundation Trust & Kings College London	K Shevket	K		Shevket
King’s College Hospital NHS Foundation Trust & Kings College London	F Speranza	F	Fabio	Speranza
King’s College Hospital NHS Foundation Trust & Kings College London	A Te	A	Amelie	Te
King's College Hospital NHS Foundation Trust & Kings College London	M Malim	M		Malim
King's College London	K Bramham	K	Kate	Bramham
King's College London	M Brown	M		Brown
King's College London	K Ismail	K	Khalida	Ismail
King's College London	T Nicholson	T	Tim	Nicholson
King's College London	C Pariante	C	Carmen	Pariante
King's College London	C Sharpe	C	Claire	Sharpe
King's College London	S Wessely	S	Simon	Wessely
King's College London	J Whitney	J		Whitney
King’s College London, British Heart Foundation Centre, London, UK & King’s College Hospital NHS Foundation Trust, London, UK	A Shah	A	Ajay	Shah
Kings College Hospital / Guys and St Thomas NHS FT	A Chiribiri	A		Chiribiri
Kings College Hospital / Guys and St Thomas NHS FT	C O'Brien	C		O'Brien
Kings College Hospital NHS Foundation Trust & Kings College London	A Hayday	A		Hayday
Leeds Teaching Hospitals & University of Leeds	A Ashworth	A	Andrew	Ashworth
Leeds Teaching Hospitals & University of Leeds	P Beirne	P	Paul	Beirne
Leeds Teaching Hospitals & University of Leeds	J Clarke	J	Jude	Clarke
Leeds Teaching Hospitals & University of Leeds	C Coupland	C		Coupland
Leeds Teaching Hospitals & University of Leeds	M Dalton	M	Matthhe	Dalton

				w	
	Leeds Teaching Hospitals & University of Leeds	C Favager	C	Clair	Favager
	Leeds Teaching Hospitals & University of Leeds	J Glossop	J	Jodie	Glossop
	Leeds Teaching Hospitals & University of Leeds	J Greenwood	J	John	Greenwood
	Leeds Teaching Hospitals & University of Leeds	L Hall	L	Lucy	Hall
	Leeds Teaching Hospitals & University of Leeds	T Hardy	T	Tim	Hardy
	Leeds Teaching Hospitals & University of Leeds	A Humphries	A	Amy	Humphries
	Leeds Teaching Hospitals & University of Leeds	J Murira	J	Jennifer	Murira
	Leeds Teaching Hospitals & University of Leeds	D Peckham	D	Dan	Peckham
	Leeds Teaching Hospitals & University of Leeds	S Plein	S		Plein
	Leeds Teaching Hospitals & University of Leeds	J Rangeley	J	Jade	Rangeley
	Leeds Teaching Hospitals & University of Leeds	G Saalmink	G	Gwen	Saalmink
	Leeds Teaching Hospitals & University of Leeds	A L Tan	A L	Ai Lyn	Tan
	Leeds Teaching Hospitals & University of Leeds	E Wade	E	Elaine	Wade
	Leeds Teaching Hospitals & University of Leeds	B Whittam	B	Beverley	Whittam
	Leeds Teaching Hospitals & University of Leeds	N Window	N	Nicola	Window
	Leeds Teaching Hospitals & University of Leeds	J Woods	J	Janet	Woods
	Lewisham & Greenwich NHS Trust	G Coakley	G		Coakley
	Liverpool University Hospitals NHS Foundation Trust & University of Liverpool	L Turtle	L	Lance	Turtle
	Liverpool University Hospitals NHS Foundation Trust & University of Liverpool	L Allerton	L	Lisa	Allerton
	Liverpool University Hospitals NHS Foundation Trust & University of Liverpool	AM Allt	AM	Ann Marie	Allt
	Liverpool University Hospitals NHS Foundation Trust & University of Liverpool	M Beadsworth	M		Beadsworth
	Liverpool University Hospitals NHS Foundation Trust & University of Liverpool	A Berridge	A	Anthony	Berridge
	Liverpool University Hospitals NHS Foundation Trust & University of Liverpool	J Brown	J	Jo	Brown
	Liverpool University Hospitals NHS Foundation Trust & University of Liverpool	S Cooper	S	Shirley	Cooper
	Liverpool University Hospitals NHS Foundation Trust & University of Liverpool	A Cross	A	Andy	Cross
	Liverpool University Hospitals NHS Foundation Trust & University of Liverpool	S Defres	S	Sylviane	Defres
	Liverpool University Hospitals NHS Foundation Trust & University of Liverpool	S L Dobson	S L		Dobson

Liverpool University Hospitals NHS Foundation Trust & University of Liverpool	J Earley	J	Joanne	Earley
Liverpool University Hospitals NHS Foundation Trust & University of Liverpool	N French	N		French
Liverpool University Hospitals NHS Foundation Trust & University of Liverpool	W Greenhalf	W	William	Greenhalf
Liverpool University Hospitals NHS Foundation Trust & University of Liverpool	K Hainey	K	Kera	Hainey
Liverpool University Hospitals NHS Foundation Trust & University of Liverpool	H E Hardwick	H E	Hayley	Hardwick
Liverpool University Hospitals NHS Foundation Trust & University of Liverpool	J Hawkes	J	Jenny	Hawkes
Liverpool University Hospitals NHS Foundation Trust & University of Liverpool	V Highett	V	Victoria	Highett
Liverpool University Hospitals NHS Foundation Trust & University of Liverpool	S Kaprowska	S	Sabina	Kaprowska
Liverpool University Hospitals NHS Foundation Trust & University of Liverpool	A L Key	A L	Angela	Key
Liverpool University Hospitals NHS Foundation Trust & University of Liverpool	L Lavelle-Langham	L	Lara	Lavelle-Langham
Liverpool University Hospitals NHS Foundation Trust & University of Liverpool	N Lewis-Burke	N		Lewis-Burke
Liverpool University Hospitals NHS Foundation Trust & University of Liverpool	G Madzamba	G	Gladys	Madzamba
Liverpool University Hospitals NHS Foundation Trust & University of Liverpool	F Malein	F	Flora	Malein
Liverpool University Hospitals NHS Foundation Trust & University of Liverpool	S Marsh	S	Sophie	Marsh
Liverpool University Hospitals NHS Foundation Trust & University of Liverpool	C Mears	C	Chloe	Mears
Liverpool University Hospitals NHS Foundation Trust & University of Liverpool	L Melling	L	Lucy	Melling
Liverpool University Hospitals NHS Foundation Trust & University of Liverpool	M J Noonan	M J	Matthew	Noonan
Liverpool University Hospitals NHS Foundation Trust & University of Liverpool	L Poll	L		Poll
Liverpool University Hospitals NHS Foundation Trust & University of Liverpool	J Pratt	J	James	Pratt
Liverpool University Hospitals NHS Foundation Trust & University of Liverpool	E Richardson	E	Emma	Richardson
Liverpool University Hospitals NHS Foundation Trust & University of Liverpool	A Rowe	A	Anna	Rowe
Liverpool University Hospitals NHS Foundation Trust & University of Liverpool	M G Semple	M G	Calum	Semple
Liverpool University Hospitals NHS Foundation Trust & University of Liverpool	V Shaw	V	Victoria	Shaw
Liverpool University Hospitals NHS Foundation Trust & University of Liverpool	K A Tripp	K A		Tripp
Liverpool University Hospitals NHS Foundation Trust & University of Liverpool	L O Wajero	L O	Lilian	Wajero
Liverpool University Hospitals NHS Foundation Trust & University of Liverpool	S A Williams-Howard	S A		Williams-Howard

	Liverpool University Hospitals NHS Foundation Trust & University of Liverpool	D G Wootton	D G	Dan	Wootton
	Liverpool University Hospitals NHS Foundation Trust & University of Liverpool	J Wyles	J		Wyles
	London North West University Healthcare NHS Trust	S N Diwanji	S N	Shalin	Diwanji
	London North West University Healthcare NHS Trust	S Gurram	S	Sambasiv arao	Gurram
	London North West University Healthcare NHS Trust	P Papineni	P	Padmasa yee	Papineni
	London North West University Healthcare NHS Trust	S Quaid	S	Sheena	Quaid
	London North West University Healthcare NHS Trust	G F Tiongson	G F	Gerlynn	Tiongson
	London North West University Healthcare NHS Trust	E Watson	E	Ekaterina	Watson
	London School of Hygiene & Tropical Medicine	A Briggs	A	Andrew	Briggs
	London School of Hygiene & Tropical Medicine	M Marks	M	Michael	Marks
	Long Covid Support	C Hastie	C	Claire	Hastie
	Long Covid Support	N Rogers	N	Natalie	Rogers
	Long Covid Support	N Smith	N	Nikki	Smith
	Loughborough University	D Stensel	D	David	Stensel
	Loughborough University	L Bishop	L	Lettie	Bishop
	Manchester Centre for Clinical Neurosciences, Salford Royal NHS Foundation Trust	K McIvor	K	Katherine	McIvor
	Manchester University NHD Foundation Trust	P Rivera-Ortega	P	Pilar	Rivera-Ortega
	Manchester University NHS Foundation Trust & University of Manchester	B Al-Sheklly	B	Bashar	Al-Sheklly
	Manchester University NHS Foundation Trust & University of Manchester	C Avram	C	Cristina	Avram
	Manchester University NHS Foundation Trust & University of Manchester	J Blaikely	J	John	Blaikely
	Manchester University NHS Foundation Trust & University of Manchester	M Buch	M		Buch
	Manchester University NHS Foundation Trust & University of Manchester	N Choudhury	N		Choudhury
	Manchester University NHS Foundation Trust & University of Manchester	D Faluyi	D	David	Faluyi
	Manchester University NHS Foundation Trust & University of Manchester	T Felton	T		Felton
	Manchester University NHS Foundation Trust & University of Manchester	T Gorsuch	T		Gorsuch
	Manchester University NHS Foundation Trust & University of Manchester	N A Hanley	N A	Neil	Hanley

Manchester University NHS Foundation Trust & University of Manchester	A Horsley	A	Alex	Horsley
Manchester University NHS Foundation Trust & University of Manchester	T Hussell	T	Tracy	Hussell
Manchester University NHS Foundation Trust & University of Manchester	Z Kausar	Z	Zunaira	Kausar
Manchester University NHS Foundation Trust & University of Manchester	N Odell	N	Natasha	Odell
Manchester University NHS Foundation Trust & University of Manchester	R Osbourne	R	Rebecca	Osbourne
Manchester University NHS Foundation Trust & University of Manchester	K Piper Hanley	K	Karen	Piper Hanley
Manchester University NHS Foundation Trust & University of Manchester	K Radhakrishnan	K		Radhakrishnan
Manchester University NHS Foundation Trust & University of Manchester	S Stockdale	S	Sue	Stockdale
McPin Foundation	T Kabir	T	Thomas	Kabir
MRC - University of Glasgow Centre for Virus Research	J T Scott	J T	Janet	Scott
National Heart and Lung Institute, Imperial College London	I D Stewart	I D	Iain	Stewart
National Heart and Lung Institute, Imperial College London, London, United Kingdom	P J M Openshaw	P J M	Peter	Openshaw
Newcastle University / Chair of NIHR Demantia TRC	D Burn	D	David	Burn
Newcastle upon Tyne Hospitals NHS Foundation Trust & University of Newcastle	A Ayoub	A		Ayoub
Newcastle upon Tyne Hospitals NHS Foundation Trust & University of Newcastle	J Brown	J		Brown
Newcastle upon Tyne Hospitals NHS Foundation Trust & University of Newcastle	G Burns	G		Burns
Newcastle upon Tyne Hospitals NHS Foundation Trust & University of Newcastle	G Davies	G	Gareth	Davies
Newcastle upon Tyne Hospitals NHS Foundation Trust & University of Newcastle	A De Soyza	A	Anthony	De Soyza
Newcastle upon Tyne Hospitals NHS Foundation Trust & University of Newcastle	C Echevarria	C	Carlos	Echevarria
Newcastle upon Tyne Hospitals NHS Foundation Trust & University of Newcastle	H Fisher	H	Helen	Fisher
Newcastle upon Tyne Hospitals NHS Foundation Trust & University of Newcastle	C Francis	C		Francis
Newcastle upon Tyne Hospitals NHS Foundation Trust & University of Newcastle	A Greenhalgh	A	Alan	Greenhalgh
Newcastle upon Tyne Hospitals NHS Foundation Trust & University of Newcastle	P Hogarth	P	Philip	Hogarth
Newcastle upon Tyne Hospitals NHS Foundation Trust & University of Newcastle	J Hughes	J	Joan	Hughes
Newcastle upon Tyne Hospitals NHS Foundation Trust & University of Newcastle	K Jiwa	K	Kasim	Jiwa
Newcastle upon Tyne Hospitals NHS Foundation Trust & University of Newcastle	G Jones	G		Jones
Newcastle upon Tyne Hospitals NHS Foundation Trust & University of Newcastle	G MacGowan	G		MacGowan

Newcastle upon Tyne Hospitals NHS Foundation Trust & University of Newcastle	D Price	D		Price
Newcastle upon Tyne Hospitals NHS Foundation Trust & University of Newcastle	A Sayer	A	Avan	Sayer
Newcastle upon Tyne Hospitals NHS Foundation Trust & University of Newcastle	J Simpson	J	John	Simpson
Newcastle upon Tyne Hospitals NHS Foundation Trust & University of Newcastle	H Tedd	H		Tedd
Newcastle upon Tyne Hospitals NHS Foundation Trust & University of Newcastle	S Thomas	S		Thomas
Newcastle upon Tyne Hospitals NHS Foundation Trust & University of Newcastle	S West	S	Sophie	West
Newcastle upon Tyne Hospitals NHS Foundation Trust & University of Newcastle	M Witham	M		Witham
Newcastle upon Tyne Hospitals NHS Foundation Trust & University of Newcastle	S Wright	S		Wright
Newcastle upon Tyne Hospitals NHS Foundation Trust & University of Newcastle	A Young	A		Young
NHS Dumfries and Galloway	M J McMahon	M J	Michael	McMahon
NHS Dumfries and Galloway	P Neill	P	Paula	Neill
NHS Greater Glasgow and Clyde Health Board & University of Glasgow	D Anderson	D	David	Anderson
NHS Greater Glasgow and Clyde Health Board & University of Glasgow	N Basu	N	Neil	Basu
NHS Greater Glasgow and Clyde Health Board & University of Glasgow	H Bayes	H	Hannah	Bayes
NHS Greater Glasgow and Clyde Health Board & University of Glasgow	A Brown	A	Ammani	Brown
NHS Greater Glasgow and Clyde Health Board & University of Glasgow	A Dougherty	A	Andrew	Dougherty
NHS Greater Glasgow and Clyde Health Board & University of Glasgow	K Fallon	K		Fallon
NHS Greater Glasgow and Clyde Health Board & University of Glasgow	L Gilmour	L		Gilmour
NHS Greater Glasgow and Clyde Health Board & University of Glasgow	D Grieve	D		Grieve
NHS Greater Glasgow and Clyde Health Board & University of Glasgow	K Mangion	K		Mangion
NHS Greater Glasgow and Clyde Health Board & University of Glasgow	A Morrow	A		Morrow
NHS Greater Glasgow and Clyde Health Board & University of Glasgow	R Sykes	R		Sykes
NHS Greater Glasgow and Clyde Health Board & University of Glasgow	C Berry	C	Colin	Berry
NHS Greater Glasgow and Clyde Health Board & University of Glasgow	I B McInnes	I B		McInnes
NHS Greater Glasgow and Clyde Health Board & University of Glasgow	K Scott	K	Kathryn	Scott
NHS Highland	F Barrett	F	Fiona	Barrett
NHS Highland	A Donaldson	A		Donaldson
NHS Highland	E K Sage	E K	Beth	Sage
NHS Lanarkshire	D Bell	M	Murdina	Bell

NHS Lanarkshire	A Brown	A	Angela	Brown
NHS Lanarkshire	M Brown	M		Brown
NHS Lanarkshire	R Hamil	R		Hamil
NHS Lanarkshire	K Leitch	K	Karen	Leitch
NHS Lanarkshire	L Macliver	L		Macliver
NHS Lanarkshire	M Patel	M	Manish	Patel
NHS Lanarkshire	J Quigley	J	Jackie	Quigley
NHS Lanarkshire	A Smith	A	Andrew	Smith
NHS Lanarkshire	B Welsh	B		Welsh
NHS Lothian & University of Edinburgh	G Choudhury	G	Gaunab	Choudhury
NHS Lothian & University of Edinburgh	S Clohisey	S		Clohisey
NHS Lothian & University of Edinburgh	A Deans	A	Andrew	Deans
NHS Lothian & University of Edinburgh	A B Docherty	A B	Annemarie	Docherty
NHS Lothian & University of Edinburgh	J Furniss	J		Furniss
NHS Lothian & University of Edinburgh	E M Harrison	E M	Ewen	Harrison
NHS Lothian & University of Edinburgh	S Kelly	S		Kelly
NHS Lothian & University of Edinburgh	A Sheikh	A	Aziz	Sheikh
NHS Tayside & University of Dundee	J D Chalmers	J D	James	Chalmers
NHS Tayside & University of Dundee	D Connell	D	David	Connell
NHS Tayside & University of Dundee	C Deas	C		Deas
NHS Tayside & University of Dundee	A Elliott	A	Anne	Elliott
NHS Tayside & University of Dundee	J George	J		George
NHS Tayside & University of Dundee	S Mohammed	S		Mohammed
NHS Tayside & University of Dundee	J Rowland	J		Rowland
NHS Tayside & University of Dundee	A R Solstice	AR		Solstice
NHS Tayside & University of Dundee	D Sutherland	D	Debbie	Sutherland
NHS Tayside & University of Dundee	C J Tee	CJ	Caroline	Tee
NIHR Leicester Biomedical Research Centre-Respiratory Patient and Public Involvement Group	J Bunker	J	Jenny	Bunker

NIHR Leicester Biomedical Research Centre-Respiratory Patient and Public Involvement Group	R Gill	R	Rhyan	Gill
NIHR Leicester Biomedical Research Centre-Respiratory Patient and Public Involvement Group	R Nathu	R	Rashmita	Nathu
NIHR Office for Clinical Research Infrastructure	K Holmes	K	Katie	Holmes
North Bristol NHS Trust & University of Bristol	H Adamali	H		Adamali
North Bristol NHS Trust & University of Bristol	D Arnold	D	David	Arnold
North Bristol NHS Trust & University of Bristol	S Barratt	S	Shaney	Barratt
North Bristol NHS Trust & University of Bristol	A Dipper	A		Dipper
North Bristol NHS Trust & University of Bristol	S Dunn	S	Sarah	Dunn
North Bristol NHS Trust & University of Bristol	N Maskell	N	Nick	Maskell
North Bristol NHS Trust & University of Bristol	A Morley	A	Anna	Morley
North Bristol NHS Trust & University of Bristol	L Morrison	L	Leigh	Morrison
North Bristol NHS Trust & University of Bristol	L Stadon	L	Louise	Stadon
North Bristol NHS Trust & University of Bristol	S Waterson	S	Samuel	Waterson
North Bristol NHS Trust & University of Bristol	H Welch	H		Welch
North Middlesex University Hospital NHS Trust	B Jayaraman	B	Bhagy	Jayaraman
North Middlesex University Hospital NHS Trust	T Light	T	Tessa	Light
Northumbria University	I Vogiatzis	I	Ioannis	Vogiatzis
Nottingham University Hospitals NHS Trust & University of Nottingham	P Almeida	P	Paula	Almeida
Nottingham University Hospitals NHS Trust & University of Nottingham	C E Bolton	C E	Charlotte	Bolton
Nottingham University Hospitals NHS Trust & University of Nottingham	A Hosseini	A	Akram	Hosseini
Nottingham University Hospitals NHS Trust & University of Nottingham	L Matthews	L	Laura	Matthews
Nottingham University Hospitals NHS Trust & University of Nottingham	R Needham	R	Robert	Needham
Nottingham University Hospitals NHS Trust & University of Nottingham	K Shaw	K	Karen	Shaw
Nottingham University Hospitals NHS Trust & University of Nottingham	A K Thomas	A K	Andrew	Thomas
Nottingham University Hospitals NHS Trust & University of Nottingham	J Bonnington	J		Bonnington
Nottingham University Hospitals NHS Trust & University of Nottingham	M Chrystal	M	Melanie	Chrystal
Nottingham University Hospitals NHS Trust & University of Nottingham	C Dupont	C	Catherine	Dupont
Nottingham University Hospitals NHS Trust & University of Nottingham	P L Greenhaff	P L	Paul	Greenhaff
Nottingham University Hospitals NHS Trust & University of Nottingham	A Gupta	A	Ayushma	Gupta

			n	
Nottingham University Hospitals NHS Trust & University of Nottingham	W Jang	W		Jang
Nottingham University Hospitals NHS Trust & University of Nottingham	S Linford	S		Linford
Nottingham University Hospitals NHS Trust & University of Nottingham	A Nikolaidis	A	Athanasio s	Nikolaidis
Nottingham University Hospitals NHS Trust & University of Nottingham	S Prosper	S	Sabrina	Prosper
Oxford University Hospitals NHS Foundation Trust	A Burns	A		Burns
Oxford University Hospitals NHS Foundation Trust	N Kanellakis	N		Kanellakis
Oxford University Hospitals NHS Foundation Trust & University of Oxford	V M Ferreira	V		Ferreira
Oxford University Hospitals NHS Foundation Trust & University of Oxford	C Nikolaidou	C		Nikolaidou
Oxford University Hospitals NHS Foundation Trust & University of Oxford	C Xie	C		Xie
Oxford University Hospitals NHS Foundation Trust & University of Oxford	M Ainsworth	M	Mark	Ainsworth
Oxford University Hospitals NHS Foundation Trust & University of Oxford	A Alamoudi	A	Asma	Alamoudi
Oxford University Hospitals NHS Foundation Trust & University of Oxford	A Bloss	A	Angela	Bloss
Oxford University Hospitals NHS Foundation Trust & University of Oxford	P Carter	P	Penny	Carter
Oxford University Hospitals NHS Foundation Trust & University of Oxford	M Cassar	M		Cassar
Oxford University Hospitals NHS Foundation Trust & University of Oxford	J Chen	J	Jin	Chen
Oxford University Hospitals NHS Foundation Trust & University of Oxford	F Conneh	F	Florence	Conneh
Oxford University Hospitals NHS Foundation Trust & University of Oxford	T Dong	T		Dong
Oxford University Hospitals NHS Foundation Trust & University of Oxford	R I Evans	R I	Ranurom anana	Evans
Oxford University Hospitals NHS Foundation Trust & University of Oxford	E Fraser	E	Emily	Fraser
Oxford University Hospitals NHS Foundation Trust & University of Oxford	J R Geddes	J R	John	Geddes
Oxford University Hospitals NHS Foundation Trust & University of Oxford	F Gleeson	F		Gleeson
Oxford University Hospitals NHS Foundation Trust & University of Oxford	P Harrison	P	Paul	Harrison
Oxford University Hospitals NHS Foundation Trust & University of Oxford	M Havinden-Williams	M	May	Havinden-Williams
Oxford University Hospitals NHS Foundation Trust & University of Oxford	L P Ho	L P	Ling Pei	Ho
Oxford University Hospitals NHS Foundation Trust & University of Oxford	P Jezzard	P		Jezzard

Oxford University Hospitals NHS Foundation Trust & University of Oxford	I Koychev	I	Ivan	Koychev
Oxford University Hospitals NHS Foundation Trust & University of Oxford	P Kurupati	P	Prathiba	Kurupati
Oxford University Hospitals NHS Foundation Trust & University of Oxford	H McShane	H		McShane
Oxford University Hospitals NHS Foundation Trust & University of Oxford	C Megson	C	Clare	Megson
Oxford University Hospitals NHS Foundation Trust & University of Oxford	S Neubauer	S	Stefan	Neubauer
Oxford University Hospitals NHS Foundation Trust & University of Oxford	D Nicoll	D	Debby	Nicoll
Oxford University Hospitals NHS Foundation Trust & University of Oxford	G Ogg	G		Ogg
Oxford University Hospitals NHS Foundation Trust & University of Oxford	E Pacpaco	E	Edmund	Pacpaco
Oxford University Hospitals NHS Foundation Trust & University of Oxford	M Pavlides	M		Pavlides
Oxford University Hospitals NHS Foundation Trust & University of Oxford	Y Peng	Y	Yanchun	Peng
Oxford University Hospitals NHS Foundation Trust & University of Oxford	N Petousi	N	Nayia	Petousi
Oxford University Hospitals NHS Foundation Trust & University of Oxford	J Pimm	J	John	Pimm
Oxford University Hospitals NHS Foundation Trust & University of Oxford	N M Rahman	N M	Najib	Rahman
Oxford University Hospitals NHS Foundation Trust & University of Oxford	B Raman	B	Betty	Raman
Oxford University Hospitals NHS Foundation Trust & University of Oxford	M J Rowland	M J		Rowland
Oxford University Hospitals NHS Foundation Trust & University of Oxford	K Saunders	K	Kathryn	Saunders
Oxford University Hospitals NHS Foundation Trust & University of Oxford	M Sharpe	M	Michael	Sharpe
Oxford University Hospitals NHS Foundation Trust & University of Oxford	N Talbot	N	Nick	Talbot
Oxford University Hospitals NHS Foundation Trust & University of Oxford	E M Tunnicliffe	E M		Tunnicliffe
Queen Mary University of London	A Korszun	A	Ania	Korszun
Roslin Institute, The University of Edinburgh, Edinburgh, EH8 9AG, UK	S Kerr	S	Steven	Kerr
Royal Brompton and Harefield Clinical Group, Guy's and St Thomas' NHS Foundation trust.	R E Barker	R E		Barker
Royal Brompton and Harefield Clinical Group, Guy's and St Thomas' NHS Foundation trust.	D Cristiano	D	Daniele	Cristiano
Royal Brompton and Harefield Clinical Group, Guy's and St Thomas' NHS Foundation trust.	N Dormand	N		Dormand
Royal Brompton and Harefield Clinical Group, Guy's and St Thomas' NHS Foundation trust.	P George	P		George
Royal Brompton and Harefield Clinical Group, Guy's and St Thomas' NHS Foundation trust.	M Gummadi	M	Mahitha	Gummadi
Royal Brompton and Harefield Clinical Group, Guy's and St Thomas' NHS Foundation trust.	S Kon	S		Kon

Royal Brompton and Harefield Clinical Group, Guy’s and St Thomas’ NHS Foundation trust.	K Liyanage	K	Kamal	Liyanage
Royal Brompton and Harefield Clinical Group, Guy’s and St Thomas’ NHS Foundation trust.	C M Nolan	C M		Nolan
Royal Brompton and Harefield Clinical Group, Guy’s and St Thomas’ NHS Foundation trust.	B Patel	B		Patel
Royal Brompton and Harefield Clinical Group, Guy’s and St Thomas’ NHS Foundation trust.	S Patel	S	Suhani	Patel
Royal Brompton and Harefield Clinical Group, Guy’s and St Thomas’ NHS Foundation trust.	O Polgar	O	Oliver	Polgar
Royal Brompton and Harefield Clinical Group, Guy’s and St Thomas’ NHS Foundation trust.	L Price	L		Price
Royal Brompton and Harefield Clinical Group, Guy’s and St Thomas’ NHS Foundation trust.	P Shah	P		Shah
Royal Brompton and Harefield Clinical Group, Guy’s and St Thomas’ NHS Foundation trust.	S Singh	S	Suver	Singh
Royal Brompton and Harefield Clinical Group, Guy’s and St Thomas’ NHS Foundation trust.	J A Walsh	J A		Walsh
Royal Devon and Exeter NHS Trust	M Gibbons	M	Michael	Gibbons
Royal Free London NHS Foundation Trust	S Ahmad	S	Shanaz	Ahmad
Royal Free London NHS Foundation Trust	S Brill	S	Simon	Brill
Royal Free London NHS Foundation Trust	J Hurst	J	John	Hurst
Royal Free London NHS Foundation Trust	H Jarvis	H	Hannah	Jarvis
Royal Free London NHS Foundation Trust	L Lim	L	Lai	Lim
Royal Free London NHS Foundation Trust	S Mandal	S		Mandal
Royal Free London NHS Foundation Trust	D Matila	D	Darwin	Matila
Royal Free London NHS Foundation Trust	O Olaosebikan	O	Olaoluwa	Olaosebikan
Royal Free London NHS Foundation Trust	C Singh	C	Claire	Singh
Royal Free London NHS Foundation Trust	C Laing	C		Laing
Royal Papworth Hospital NHS Foundation Trust	H Baxendale	H	Helen	Baxendale
Royal Papworth Hospital NHS Foundation Trust	L Garner	L	Lucie	Garner
Royal Papworth Hospital NHS Foundation Trust	C Johnson	C		Johnson
Royal Papworth Hospital NHS Foundation Trust	J Mackie	J		Mackie
Royal Papworth Hospital NHS Foundation Trust	A Michael	A	Alice	Michael
Royal Papworth Hospital NHS Foundation Trust	J Newman	J		Newman
Royal Papworth Hospital NHS Foundation Trust	J Pack	J	Jamie	Pack
Royal Papworth Hospital NHS Foundation Trust	K Paques	K		Paques
Royal Papworth Hospital NHS Foundation Trust	H Parfrey	H		Parfrey

	Royal Papworth Hospital NHS Foundation Trust	J Parmar	J		Parmar
	Royal Papworth Hospital NHS Foundation Trust	A Reddy	A		Reddy
	Royal Surrey NHS Foundation Trust	M Halling-Brown	M	Mark	Halling-Brown
	Salford Royal NHS Foundation Trust	P Dark	P		Dark
	Salford Royal NHS Foundation Trust	N Diar-Bakerly	N	Nawar	Diar-Bakerly
	Salford Royal NHS Foundation Trust	D Evans	D		Evans
	Salford Royal NHS Foundation Trust	E Hardy	E		Hardy
	Salford Royal NHS Foundation Trust	A Harvey	A	Alice	Harvey
	Salford Royal NHS Foundation Trust	D Holgate	D		Holgate
	Salford Royal NHS Foundation Trust	S Knight	S	Sean	Knight
	Salford Royal NHS Foundation Trust	N Mairs	N		Mairs
	Salford Royal NHS Foundation Trust	N Majeed	N		Majeed
	Salford Royal NHS Foundation Trust	L McMorrow	L		McMorrow
	Salford Royal NHS Foundation Trust	J Oxton	J		Oxton
	Salford Royal NHS Foundation Trust	J Pendlebury	J	Jessica	Pendlebury
	Salford Royal NHS Foundation Trust	C Summersgill	C		Summersgill
	Salford Royal NHS Foundation Trust	R Ugwuoke	R		Ugwuoke
	Salford Royal NHS Foundation Trust	S Whittaker	S		Whittaker
	Salisbury NHS Foundation Trust	W Matimba-Mupaya	W	Wadzanai	Matimba-Mupaya
	Salisbury NHS Foundation Trust	S Strong-Sheldrake	S	Sophia	Strong-Sheldrake
	School of Cardiovascular Medicine & Sciences. King's College London	P Chowienczyk	P	Phillip	Chowienczyk
	Sheffield Teaching NHS Foundation Trust & University of Sheffield	J Bagshaw	J		Bagshaw
	Sheffield Teaching NHS Foundation Trust & University of Sheffield	M Begum	M		Begum
	Sheffield Teaching NHS Foundation Trust & University of Sheffield	K Birchall	K		Birchall
	Sheffield Teaching NHS Foundation Trust & University of Sheffield	R Butcher	R	Robyn	Butcher
	Sheffield Teaching NHS Foundation Trust & University of Sheffield	H Carborn	H		Carborn

	Sheffield Teaching NHS Foundation Trust & University of Sheffield	F Chan	F	Flora	Chan
	Sheffield Teaching NHS Foundation Trust & University of Sheffield	K Chapman	K	Kerry	Chapman
	Sheffield Teaching NHS Foundation Trust & University of Sheffield	Y Cheng	Y	Yutung	Cheng
	Sheffield Teaching NHS Foundation Trust & University of Sheffield	L Chetham	L	Luke	Chetham
	Sheffield Teaching NHS Foundation Trust & University of Sheffield	C Clark	C	Cameron	Clark
	Sheffield Teaching NHS Foundation Trust & University of Sheffield	Z Coburn	Z	Zach	Coburn
	Sheffield Teaching NHS Foundation Trust & University of Sheffield	J Cole	J	Joby	Cole
	Sheffield Teaching NHS Foundation Trust & University of Sheffield	M Dixon	M	Myles	Dixon
	Sheffield Teaching NHS Foundation Trust & University of Sheffield	A Fairman	A	Alexandra	Fairman
	Sheffield Teaching NHS Foundation Trust & University of Sheffield	J Finnigan	J		Finnigan
	Sheffield Teaching NHS Foundation Trust & University of Sheffield	H Foot	H		Foot
	Sheffield Teaching NHS Foundation Trust & University of Sheffield	D Foote	D	David	Foote
	Sheffield Teaching NHS Foundation Trust & University of Sheffield	A Ford	A	Amber	Ford
	Sheffield Teaching NHS Foundation Trust & University of Sheffield	R Gregory	R	Rebecca	Gregory
	Sheffield Teaching NHS Foundation Trust & University of Sheffield	K Harrington	K	Kate	Harrington
	Sheffield Teaching NHS Foundation Trust & University of Sheffield	L Haslam	L		Haslam
	Sheffield Teaching NHS Foundation Trust & University of Sheffield	L Hesselden	L		Hesselden
	Sheffield Teaching NHS Foundation Trust & University of Sheffield	J Hockridge	J		Hockridge
	Sheffield Teaching NHS Foundation Trust & University of Sheffield	A Holbourn	A	Ailsa	Holbourn
	Sheffield Teaching NHS Foundation Trust & University of Sheffield	B Holroyd-Hind	B		Holroyd-Hind
	Sheffield Teaching NHS Foundation Trust & University of Sheffield	L Holt	L		Holt
	Sheffield Teaching NHS Foundation Trust & University of Sheffield	A Howell	A	Alice	Howell
	Sheffield Teaching NHS Foundation Trust & University of Sheffield	E Hurditch	E		Hurditch
	Sheffield Teaching NHS Foundation Trust & University of Sheffield	F Ilyas	F		Ilyas
	Sheffield Teaching NHS Foundation Trust & University of Sheffield	C Jarman	C	Claire	Jarman
	Sheffield Teaching NHS Foundation Trust & University of Sheffield	A Lawrie	A	Allan	Lawrie
	Sheffield Teaching NHS Foundation Trust & University of Sheffield	J-H Lee	J-H	Ju Hee	Lee
	Sheffield Teaching NHS Foundation Trust & University of Sheffield	E Lee	E	Elvina	Lee
	Sheffield Teaching NHS Foundation Trust & University of Sheffield	R Lenagh	R	Rebecca	Lenagh

Sheffield Teaching NHS Foundation Trust & University of Sheffield	A Lye	A	Alison	Lye
Sheffield Teaching NHS Foundation Trust & University of Sheffield	I Macharia	I	Irene	Macharia
Sheffield Teaching NHS Foundation Trust & University of Sheffield	M Marshall	M		Marshall
Sheffield Teaching NHS Foundation Trust & University of Sheffield	A Mbuyisa	A	Angeline	Mbuyisa
Sheffield Teaching NHS Foundation Trust & University of Sheffield	J McNeill	J		McNeill
Sheffield Teaching NHS Foundation Trust & University of Sheffield	S Megson	S	Sharon	Megson
Sheffield Teaching NHS Foundation Trust & University of Sheffield	J Meiring	J		Meiring
Sheffield Teaching NHS Foundation Trust & University of Sheffield	L Milner	L		Milner
Sheffield Teaching NHS Foundation Trust & University of Sheffield	S Misra	S		Misra
Sheffield Teaching NHS Foundation Trust & University of Sheffield	H Newell	H	Helen	Newell
Sheffield Teaching NHS Foundation Trust & University of Sheffield	T Newman	T	Tom	Newman
Sheffield Teaching NHS Foundation Trust & University of Sheffield	C Norman	C		Norman
Sheffield Teaching NHS Foundation Trust & University of Sheffield	L Nwafor	L	Lorenza	Nwafor
Sheffield Teaching NHS Foundation Trust & University of Sheffield	D Pattenadk	D	Dibya	Pattenadk
Sheffield Teaching NHS Foundation Trust & University of Sheffield	M Plowright	M	Megan	Plowright
Sheffield Teaching NHS Foundation Trust & University of Sheffield	J Porter	J	Julie	Porter
Sheffield Teaching NHS Foundation Trust & University of Sheffield	P Ravencroft	P	Phillip	Ravencroft
Sheffield Teaching NHS Foundation Trust & University of Sheffield	C Roddis	C		Roddis
Sheffield Teaching NHS Foundation Trust & University of Sheffield	J Rodger	J		Rodger
Sheffield Teaching NHS Foundation Trust & University of Sheffield	S L Rowland-Jones	S L	Sarah	Rowland-Jones
Sheffield Teaching NHS Foundation Trust & University of Sheffield	P Saunders	P	Peter	Saunders
Sheffield Teaching NHS Foundation Trust & University of Sheffield	J Sidebottom	J		Sidebottom
Sheffield Teaching NHS Foundation Trust & University of Sheffield	J Smith	J	Jacqui	Smith
Sheffield Teaching NHS Foundation Trust & University of Sheffield	L Smith	L	Laurie	Smith
Sheffield Teaching NHS Foundation Trust & University of Sheffield	N Steele	N		Steele
Sheffield Teaching NHS Foundation Trust & University of Sheffield	G Stephens	G		Stephens
Sheffield Teaching NHS Foundation Trust & University of Sheffield	R Stimpson	R		Stimpson
Sheffield Teaching NHS Foundation Trust & University of Sheffield	B Thamu	B		Thamu

Sheffield Teaching NHS Foundation Trust & University of Sheffield	AAR Thompson	A A R	A. A. Roger	Thompson
Sheffield Teaching NHS Foundation Trust & University of Sheffield	N Tinker	N		Tinker
Sheffield Teaching NHS Foundation Trust & University of Sheffield	K Turner	K	Kim	Turner
Sheffield Teaching NHS Foundation Trust & University of Sheffield	H Turton	H	Helena	Turton
Sheffield Teaching NHS Foundation Trust & University of Sheffield	P Wade	P	Phillip	Wade
Sheffield Teaching NHS Foundation Trust & University of Sheffield	S Walker	S		Walker
Sheffield Teaching NHS Foundation Trust & University of Sheffield	J Watson	J	James	Watson
Sheffield Teaching NHS Foundation Trust & University of Sheffield	I Wilson	I	Imogen	Wilson
Sheffield Teaching NHS Foundation Trust & University of Sheffield	A Zawia	A	Amira	Zawia
Sherwood Forest Hospitals NHS Foundation Trust	L Allsop	L	Lynne	Allsop
Sherwood Forest Hospitals NHS Foundation Trust	K Bennett	K	Kaytie	Bennett
Sherwood Forest Hospitals NHS Foundation Trust	P Buckley	P	Phil	Buckley
Sherwood Forest Hospitals NHS Foundation Trust	M Flynn	M	Margaret	Flynn
Sherwood Forest Hospitals NHS Foundation Trust	M Gill	M	Mandy	Gill
Sherwood Forest Hospitals NHS Foundation Trust	C Goodwin	C	Camelia	Goodwin
Sherwood Forest Hospitals NHS Foundation Trust	M Greatorex	M		Greatorex
Sherwood Forest Hospitals NHS Foundation Trust	H Gregory	H	Heidi	Gregory
Sherwood Forest Hospitals NHS Foundation Trust	C Heeley	C	Cheryl	Heeley
Sherwood Forest Hospitals NHS Foundation Trust	L Holloway	L	Leah	Holloway
Sherwood Forest Hospitals NHS Foundation Trust	M Holmes	M	Megan	Holmes
Sherwood Forest Hospitals NHS Foundation Trust	J Hutchinson	J	John	Hutchinson
Sherwood Forest Hospitals NHS Foundation Trust	J Kirk	J	Jill	Kirk
Sherwood Forest Hospitals NHS Foundation Trust	W Lovegrove	W	Wayne	Lovegrove
Sherwood Forest Hospitals NHS Foundation Trust	TA Sewell	T A	Terri Ann	Sewell
Sherwood Forest Hospitals NHS Foundation Trust	S Shelton	S	Sarah	Shelton
Sherwood Forest Hospitals NHS Foundation Trust	D Sissons	D		Sissons
Sherwood Forest Hospitals NHS Foundation Trust	K Slack	K	Katie	Slack
Sherwood Forest Hospitals NHS Foundation Trust	S Smith	S	Susan	Smith

Sherwood Forest Hospitals NHS Foundation Trust	D Sowter	D		Sowter
Sherwood Forest Hospitals NHS Foundation Trust	S Turner	S	Sarah	Turner
Sherwood Forest Hospitals NHS Foundation Trust	V Whitworth	V		Whitworth
Sherwood Forest Hospitals NHS Foundation Trust	I Wynter	I	Inez	Wynter
Shropshire Community Health NHS Trust	J Tomlinson	J	Johanne	Tomlinson
Shropshire Community Health NHS Trust	L Warburton	L	Louise	Warburton
Shropshire Community Health NHS Trust	S Painter	S	Sharon	Painter
Somerset NHS Foundation Trust	S Palmer	S	Sue	Palmer
Somerset NHS Foundation Trust	D Redwood	D	Dawn	Redwood
Somerset NHS Foundation Trust	J Tilley	J	Jo	Tilley
Somerset NHS Foundation Trust	C Vickers	C	Carinna	Vickers
Somerset NHS Foundation Trust	T Wainwright	T	Tania	Wainwright
South London and Maudsley NHS Foundation Trust & Kings College London	G Breen	G		Breen
South London and Maudsley NHS Foundation Trust & Kings College London	M Hotopf	M		Hotopf
St George's University Hospitals NHS Foundation Trust	R Aul	R	Raminder	Aul
St George's University Hospitals NHS Foundation Trust	D Forton	D		Forton
St George’s University Hospitals NHS Foundation Trust	M Ali	M	Mariam	Ali
St George’s University Hospitals NHS Foundation Trust	A Dunleavy	A		Dunleavy
St George’s University Hospitals NHS Foundation Trust	M Mencias	M	Mark	Mencias
St George’s University Hospitals NHS Foundation Trust	N Msimanga	N		Msimanga
St George’s University Hospitals NHS Foundation Trust	T Samakomva	T		Samakomva
St George’s University Hospitals NHS Foundation Trust	S Siddique	S	Sulman	Siddique
St George’s University Hospitals NHS Foundation Trust	V Tavoukjian	V	Vera	Tavoukjian
St George’s University Hospitals NHS Foundation Trust	J Teixeira	J		Teixeira
Stroke Association	R Ahmed	R	Rubina	Ahmed
Stroke Association	R Francis	R	Richard	Francis
Swansea Bay University Health Board	L Connor	L	Lynda	Connor
Swansea Bay University Health Board	A Cook	A	Amanda	Cook
Swansea Bay University Health Board	G A Davies	G A	Gwyneth	Davies

Swansea Bay University Health Board	T Rees	T	Tabitha	Rees
Swansea Bay University Health Board	F Thaivalappil	F	Favas	Thaivalappil
Swansea Bay University Health Board	C Thomas	C	Caradog	Thomas
Swansea University	M McNarry	M		McNarry
Swansea University & Swansea Welsh Network	N Williams	N		Williams
Swansea University, Swansea Welsh Network, Hywel Dda University Health Board	K E Lewis	K E	Keir	Lewis
Tameside and Glossop Integrated Care NHS Foundation Trust	M Coulding	M	Martina	Coulding
Tameside and Glossop Integrated Care NHS Foundation Trust	H Jones	H	Heather	Jones
Tameside and Glossop Integrated Care NHS Foundation Trust	S Kilroy	S	Susan	Kilroy
Tameside and Glossop Integrated Care NHS Foundation Trust	J McCormick	J	Jacqueline	McCormick
Tameside and Glossop Integrated Care NHS Foundation Trust	J McIntosh	J	Jerome	McIntosh
Tameside and Glossop Integrated Care NHS Foundation Trust	V Turner	V	Victoria	Turner
Tameside and Glossop Integrated Care NHS Foundation Trust	J Vere	J	Joanne	Vere
Tameside and Glossop Integrated Care NHS Foundation Trust	A Butt	A	Al-Tahoor	Butt
Tameside and Glossop Integrated Care NHS Foundation Trust	H Savill	H	Heather	Savill
The Hillingdon Hospitals NHS Foundation Trust	S S Kon	S S	Samantha	Kon
The Hillingdon Hospitals NHS Foundation Trust	G Landers	G		Landers
The Hillingdon Hospitals NHS Foundation Trust	H Lota	H	Harpreet	Lota
The Hillingdon Hospitals NHS Foundation Trust	S Portukhay	S	Sofiya	Portukhay
The Hillingdon Hospitals NHS Foundation Trust	M Nasseri	M	Mariam	Nasseri
The Rotherham NHS Foundation Trust	A Daniels	A	Alison	Daniels
The Rotherham NHS Foundation Trust	A Hormis	A	Anil	Hormis
The Rotherham NHS Foundation Trust	J Ingham	J	Julie	Ingham
The Rotherham NHS Foundation Trust	L Zeidan	L	Lisa	Zeidan
United Lincolnshire Hospitals NHS Trust	M Chablani	M	Manish	Chablani
United Lincolnshire Hospitals NHS Trust	L Osborne	L	Lynn	Osborne
University College London	S Aslani	S	Shahab	Aslani
University College London	A Banerjee	A	Amita	Banerjee

	University College London	R Batterham	R		Batterham
	University College London	G Baxter	G	Gabrielle	Baxter
	University College London	R Bell	R	Robert	Bell
	University College London	A David	A	Anthony	David
	University College London	E Denneny	E	Emma	Denneny
	University College London	A D Hughes	A D	Alun	Hughes
	University College London	W Lilaonitkul	W		Lilaonitkul
	University College London	P Mehta	P		Mehta
	University College London	A Pakzad	A	Ashkan	Pakzad
	University College London	B Rangelov	B	Bojidar	Rangelov
	University College London	B Williams	B		Williams
	University College London	J Willoughby	J	James	Willoughby
	University College London	M Xu	M	Mouchen g	Xu
	University College London Hospital & University College London	N Ahwireng	N	Nyarko	Ahwireng
	University College London Hospital & University College London	D Bang	D	Dongchu n	Bang
	University College London Hospital & University College London	D Basire	D	Donna	Basire
	University College London Hospital & University College London	J S Brown	J S	Jeremy	Brown
	University College London Hospital & University College London	R C Chambers	R C	Rachel	Chambers
	University College London Hospital & University College London	A Checkley	A		Checkley
	University College London Hospital & University College London	R Evans	R		Evans
	University College London Hospital & University College London	M Heightman	M		Heightman
	University College London Hospital & University College London	T Hillman	T		Hillman
	University College London Hospital & University College London	J Jacob	J	Joseph	Jacob
	University College London Hospital & University College London	R Jastrub	R	Roman	Jastrub
	University College London Hospital & University College London	M Lipman	M		Lipman
	University College London Hospital & University College London	S Logan	S		Logan
	University College London Hospital & University College London	D Lomas	D		Lomas

University College London Hospital & University College London	M Merida Morillas	M	Marta	Merida Morillas
University College London Hospital & University College London	H Plant	H	Hannah	Plant
University College London Hospital & University College London	J C Porter	J C	Joanna	Porter
University College London Hospital & University College London	K Roy	K		Roy
University College London Hospital & University College London	E Wall	E		Wall
University College London NHS Foundation Trust, London & Barts Health NHS Trust, London, UK	T Treibel	T		Treibel
University Hospital Birmingham NHS Foundation Trust & University of Birmingham	N Ahmad Haider	N		Ahmad Haider
University Hospital Birmingham NHS Foundation Trust & University of Birmingham	C Atkin	C	Catherine	Atkin
University Hospital Birmingham NHS Foundation Trust & University of Birmingham	R Baggott	R	Rhiannon	Baggott
University Hospital Birmingham NHS Foundation Trust & University of Birmingham	M Bates	M	Michelle	Bates
University Hospital Birmingham NHS Foundation Trust & University of Birmingham	A Botkai	A		Botkai
University Hospital Birmingham NHS Foundation Trust & University of Birmingham	A Casey	A	Anna	Casey
University Hospital Birmingham NHS Foundation Trust & University of Birmingham	B Cooper	B		Cooper
University Hospital Birmingham NHS Foundation Trust & University of Birmingham	J Dasgin	J	Joanne	Dasgin
University Hospital Birmingham NHS Foundation Trust & University of Birmingham	C Dawson	C	Camilla	Dawson
University Hospital Birmingham NHS Foundation Trust & University of Birmingham	K Draxlbauer	K	Katharine	Draxlbauer
University Hospital Birmingham NHS Foundation Trust & University of Birmingham	N Gautam	N		Gautam
University Hospital Birmingham NHS Foundation Trust & University of Birmingham	J Hazeldine	J		Hazeldine
University Hospital Birmingham NHS Foundation Trust & University of Birmingham	T Hiwot	T		Hiwot
University Hospital Birmingham NHS Foundation Trust & University of Birmingham	S Holden	S	Sophie	Holden
University Hospital Birmingham NHS Foundation Trust & University of Birmingham	K Isaacs	K	Karen	Isaacs
University Hospital Birmingham NHS Foundation Trust & University of Birmingham	T Jackson	T		Jackson
University Hospital Birmingham NHS Foundation Trust & University of Birmingham	V Kamwa	V	Vicky	Kamwa
University Hospital Birmingham NHS Foundation Trust & University of Birmingham	D Lewis	D		Lewis
University Hospital Birmingham NHS Foundation Trust & University of Birmingham	J M Lord	J M	Janet	Lord
University Hospital Birmingham NHS Foundation Trust & University of Birmingham	S Madathil	S		Madathil
University Hospital Birmingham NHS Foundation Trust & University of Birmingham	C McGhee	C		McGee

University Hospital Birmingham NHS Foundation Trust & University of Birmingham	K Mcgee	K		Mcgee
University Hospital Birmingham NHS Foundation Trust & University of Birmingham	A Neal	A	Aoife	Neal
University Hospital Birmingham NHS Foundation Trust & University of Birmingham	A Newton-Cox	A	Alex	Newton-Cox
University Hospital Birmingham NHS Foundation Trust & University of Birmingham	J Nyaboko	J	Joseph	Nyaboko
University Hospital Birmingham NHS Foundation Trust & University of Birmingham	D Parekh	D	Dhruv	Parekh
University Hospital Birmingham NHS Foundation Trust & University of Birmingham	Z Peterkin	Z		Peterkin
University Hospital Birmingham NHS Foundation Trust & University of Birmingham	H Qureshi	H		Qureshi
University Hospital Birmingham NHS Foundation Trust & University of Birmingham	L Ratcliffe	L	Liz	Ratcliffe
University Hospital Birmingham NHS Foundation Trust & University of Birmingham	E Sapey	E	Elizabeth	Sapey
University Hospital Birmingham NHS Foundation Trust & University of Birmingham	J Short	J		Short
University Hospital Birmingham NHS Foundation Trust & University of Birmingham	T Soulsby	T	Tracy	Soulsby
University Hospital Birmingham NHS Foundation Trust & University of Birmingham	J Stockley	J		Stockley
University Hospital Birmingham NHS Foundation Trust & University of Birmingham	Z Suleiman	Z	Zehra	Suleiman
University Hospital Birmingham NHS Foundation Trust & University of Birmingham	T Thompson	T	Tamika	Thompson
University Hospital Birmingham NHS Foundation Trust & University of Birmingham	M Ventura	M	Maximina	Ventura
University Hospital Birmingham NHS Foundation Trust & University of Birmingham	S Walder	S	Sinead	Walder
University Hospital Birmingham NHS Foundation Trust & University of Birmingham	C Welch	C	Carly	Welch
University Hospital Birmingham NHS Foundation Trust & University of Birmingham	D Wilson	D	Daisy	Wilson
University Hospital Birmingham NHS Foundation Trust & University of Birmingham	S Yasmin	S		Yasmin
University Hospital Birmingham NHS Foundation Trust & University of Birmingham	K P Yip	K P	Kay Por	Yip
University Hospital of South Manchester NHS Foundation Trust	N Chaudhuri	N		Chaudhuri
University Hospital Southampton NHS Foundation Trust & University of Southampton	C Childs	C	Caroline	Childs
University Hospital Southampton NHS Foundation Trust & University of Southampton	R Djukanovic	R		Djukanovic
University Hospital Southampton NHS Foundation Trust & University of Southampton	S Fletcher	S		Fletcher
University Hospital Southampton NHS Foundation Trust & University of Southampton	M Harvey	M	Matt	Harvey
University Hospital Southampton NHS Foundation Trust & University of Southampton	M G Jones	M G	Mark	Jones
University Hospital Southampton NHS Foundation Trust & University of Southampton	E Marouzet	E	Elizabeth	Marouzet
University Hospital Southampton NHS Foundation Trust & University of Southampton	B Marshall	B		Marshall

University Hospital Southampton NHS Foundation Trust & University of Southampton	R Samuel	R	Reena	Samuel
University Hospital Southampton NHS Foundation Trust & University of Southampton	T Sass	T		Sass
University Hospital Southampton NHS Foundation Trust & University of Southampton	T Wallis	T	Tim	Wallis
University Hospital Southampton NHS Foundation Trust & University of Southampton	H Wheeler	H	Helen	Wheeler
University Hospitals Birmingham NHS Foundation Trust & University of Birmingham	R Steeds	R		Steeds
University Hospitals of Derby and Burton	P Beckett	P	Paul	Beckett
University Hospitals of Derby and Burton	C Dickens	C	Caroline	Dickens
University Hospitals of Derby and Burton	U Nanda	U	Uttam	Nanda
University Hospitals of Leicester NHS Trust & University of Leicester	M Aljaroof	M		Aljaroof
University Hospitals of Leicester NHS Trust & University of Leicester	N Armstrong	N	Natalie	Armstrong
University Hospitals of Leicester NHS Trust & University of Leicester	H Arnold	H		Arnold
University Hospitals of Leicester NHS Trust & University of Leicester	H Aung	H	Hnin	Aung
University Hospitals of Leicester NHS Trust & University of Leicester	M Bakali	M	Majda	Bakali
University Hospitals of Leicester NHS Trust & University of Leicester	M Bakau	M		Bakau
University Hospitals of Leicester NHS Trust & University of Leicester	E Baldry	E		Baldry
University Hospitals of Leicester NHS Trust & University of Leicester	M Baldwin	M	Molly	Baldwin
University Hospitals of Leicester NHS Trust & University of Leicester	C Bourne	C	Charlotte	Bourne
University Hospitals of Leicester NHS Trust & University of Leicester	M Bourne	M	Michelle	Bourne
University Hospitals of Leicester NHS Trust & University of Leicester	C E Brightling	C E	Chris	Brightling
University Hospitals of Leicester NHS Trust & University of Leicester	N Brunskill	N	Nigel	Brunskill
University Hospitals of Leicester NHS Trust & University of Leicester	P Cairns	P		Cairns
University Hospitals of Leicester NHS Trust & University of Leicester	L Carr	L	Liesel	Carr
University Hospitals of Leicester NHS Trust & University of Leicester	A Charalambou	A	Amanda	Charalambou
University Hospitals of Leicester NHS Trust & University of Leicester	C Christie	C		Christie
University Hospitals of Leicester NHS Trust & University of Leicester	M J Davies	M J	Melanie	Davies
University Hospitals of Leicester NHS Trust & University of Leicester	E Daynes	E	Enya	Daynes
University Hospitals of Leicester NHS Trust & University of Leicester	S Diver	S	Sarah	Diver
University Hospitals of Leicester NHS Trust & University of Leicester	R Dowling	R	Rachael	Dowling
University Hospitals of Leicester NHS Trust & University of Leicester	S Edwards	S	Sarah	Edwards

	University Hospitals of Leicester NHS Trust & University of Leicester	C Edwardson	C		Edwardson
	University Hospitals of Leicester NHS Trust & University of Leicester	O Elneima	O	Omer	Elneima
	University Hospitals of Leicester NHS Trust & University of Leicester	H Evans	H		Evans
	University Hospitals of Leicester NHS Trust & University of Leicester	R A Evans	R A	Rachael	Evans
	University Hospitals of Leicester NHS Trust & University of Leicester	J Finch	J		Finch
	University Hospitals of Leicester NHS Trust & University of Leicester	S Glover	S	Sarah	Glover
	University Hospitals of Leicester NHS Trust & University of Leicester	N Goodman	N	Nicola	Goodman
	University Hospitals of Leicester NHS Trust & University of Leicester	B Gooptu	B	Bibek	Gooptu
	University Hospitals of Leicester NHS Trust & University of Leicester	N J Greening	N J	Neil	Greening
	University Hospitals of Leicester NHS Trust & University of Leicester	K Hadley	K	Kate	Hadley
	University Hospitals of Leicester NHS Trust & University of Leicester	P Haldar	P	Pranab	Haldar
	University Hospitals of Leicester NHS Trust & University of Leicester	B Hargadon	B	Beverley	Hargadon
	University Hospitals of Leicester NHS Trust & University of Leicester	V C Harris	V C	Victoria	Harris
	University Hospitals of Leicester NHS Trust & University of Leicester	L Houchen-Wolloff	L	Linzy	Houchen-Wolloff
	University Hospitals of Leicester NHS Trust & University of Leicester	W Ibrahim	W		Ibrahim
	University Hospitals of Leicester NHS Trust & University of Leicester	L Ingram	L		Ingram
	University Hospitals of Leicester NHS Trust & University of Leicester	K Khunti	K	Kamlesh	Khunti
	University Hospitals of Leicester NHS Trust & University of Leicester	A Lea	A		Lea
	University Hospitals of Leicester NHS Trust & University of Leicester	D Lee	D		Lee
	University Hospitals of Leicester NHS Trust & University of Leicester	H J C McAuley	H J C	Hamish	McAuley
	University Hospitals of Leicester NHS Trust & University of Leicester	G P McCann	G P	Gerry	McCann
	University Hospitals of Leicester NHS Trust & University of Leicester	P McCourt	P		McCourt
	University Hospitals of Leicester NHS Trust & University of Leicester	T Mcnally	T	Teresa	Mcnally
	University Hospitals of Leicester NHS Trust & University of Leicester	G Mills	G	George	Mills
	University Hospitals of Leicester NHS Trust & University of Leicester	W Monteiro	W	Will	Monteiro
	University Hospitals of Leicester NHS Trust & University of Leicester	M Pareek	M	Manish	Pareek
	University Hospitals of Leicester NHS Trust & University of Leicester	S Parker	S		Parker

University Hospitals of Leicester NHS Trust & University of Leicester	A Prickett	A	Anne	Prickett
University Hospitals of Leicester NHS Trust & University of Leicester	I N Qureshi	I N		Qureshi
University Hospitals of Leicester NHS Trust & University of Leicester	A Rowland	A		Rowland
University Hospitals of Leicester NHS Trust & University of Leicester	R Russell	R	Richard	Russell
University Hospitals of Leicester NHS Trust & University of Leicester	M Sereno	M	Marco	Sereno
University Hospitals of Leicester NHS Trust & University of Leicester	A Shikotra	A	Aarti	Shikotra
University Hospitals of Leicester NHS Trust & University of Leicester	S Siddiqui	S	Salman	Siddiqui
University Hospitals of Leicester NHS Trust & University of Leicester	A Singapuri	A	Ananga	Singapuri
University Hospitals of Leicester NHS Trust & University of Leicester	S J Singh	S J	Sally	Singh
University Hospitals of Leicester NHS Trust & University of Leicester	J Skeemer	J		Skeemer
University Hospitals of Leicester NHS Trust & University of Leicester	M Soares	M		Soares
University Hospitals of Leicester NHS Trust & University of Leicester	E Stringer	E		Stringer
University Hospitals of Leicester NHS Trust & University of Leicester	T Thornton	T		Thornton
University Hospitals of Leicester NHS Trust & University of Leicester	M Tobin	M	Martin	Tobin
University Hospitals of Leicester NHS Trust & University of Leicester	T J C Ward	T J C		Ward
University Hospitals of Leicester NHS Trust & University of Leicester	F Woodhead	F		Woodhead
University Hospitals of Leicester NHS Trust & University of Leicester	T Yates	T	Tom	Yates
University Hospitals of Leicester NHS Trust & University of Leicester	A J Yousuf	A J		Yousuf
University of Birmingham	M Broome	M	Mattew	Broome
University of Birmingham	P McArdle	P	Paul	McArdle
University of Birmingham	D Thickett	D	David	Thickett
University of Birmingham	R Upthegrove	R	Rachel	Upthegrove
University of Birmingham	D Wilkinson	D	Dan	Wilkinson
University of Birmingham	P Moss	P	Paul	Moss
University of Birmingham	D Wraith	D	David	Wraith
University of Bristol	J Evans	J	Jonathon	Evans
University of Cambridge	E Bullmore	E	Ed	Bullmore
University of Cambridge	J L Heeney	J L	Jonathon	Heeney

University of Cambridge	C Langenberg	C	Claudia	Langenberg
University of Cambridge	W Schwaeble	W	William	Schwaeble
University of Cambridge	C Summers	C	Charlotte	Summers
University of Cambridge	J Weir McCall	J		Weir McCall
University of Edinburgh	D Adeloye	D	Davies	Adeloye
University of Edinburgh	D E Newby	D E		Newby
University of Edinburgh	R Pius	R	Riinu	Pius
University of Edinburgh	I Rudan	I	Igor	Rudan
University of Edinburgh	M Shankar-Hari	M	Manu	Shankar-Hari
University of Edinburgh	C L Sudlow	C L	Catherine	Sudlow
University of Edinburgh	M Thorpe	M	Mat	Thorpe
University of Edinburgh	S Walmsley	S	Sarah	Walmsley
University of Edinburgh	B Zheng	B	Bang	Zheng
University of Exeter	L Allan	L	Louise	Allan
University of Exeter	C Ballard	C	Clive	Ballard
University of Exeter	A McGovern	A	Andrew	McGovern
University of Exeter Medical School	J Dennis	J		Dennis
University of Glasgow	J Cavanagh	J	Jonathon	Cavanagh
University of Glasgow	S MacDonald	S		MacDonald
University of Glasgow	K O'Donnell	K	Kate	O'Donnell
University of Glasgow	J Petrie	J	John	Petrie
University of Glasgow	N Sattar	N	Naveed	Sattar
University of Glasgow	M Spears	M	Mark	Spears
University of Leeds	E Guthrie	E	Elspeth	Guthrie
University of Leeds	M Henderson	M	Max	Henderson
University of Leicester	R J Allen	R J	Richard	Allen
University of Leicester	M Bingham	M	Michelle	Bingham
University of Leicester	T Brugha	T	Terry	Brugha

University of Leicester	S Finney	S	Selina	Finney
University of Leicester	R Free	R	Rob	Free
University of Leicester	D Jones	D	Don	Jones
University of Leicester	C Lawson	C	Claire	Lawson
University of Leicester	L Gardiner	L	Gardiner	Lucy
University of Leicester	A J Moss	A J	Alistair	Moss
University of Leicester	E Mukaetova-Ladinska	E	Elizabeta	Mukaetova-Ladinska
University of Leicester	P Novotny	P	Petr	Novotny
University of Leicester	C Overton	C	Charlotte	Overton
University of Leicester	J E Pearl	J E	John	Pearl
University of Leicester	T Plekhanova	T	Tatiana	Plekhanova
University of Leicester	M Richardson	M		Richardson
University of Leicester	N Samani	N	Nilesh	Samani
University of Leicester	J Sargent	J	Jack	Sargant
University of Leicester	M Sharma	M		Sharma
University of Leicester	M Steiner	M	Mike	Steiner
University of Leicester	C Taylor	C	Chris	Taylor
University of Leicester	S Terry	S	Sarah	Terry
University of Leicester	C Tong	C		Tong
University of Leicester	E Turner	E		Turner
University of Leicester	J Wormleighton	J		Wormleighton
University of Leicester	B Zhao	B	Bang	Zhao
University of Leicester	K Ntotsis	K	Kimon	Ntotsis
University of Leicester	R M Saunders	R M	Ruth	Saunders
University of Leicester	D Lozano-Rojas	D	Daniel	Lozano-Rojas
University of Leicester	A F Goemans	A F	Anne	Goemans
University of Liverpool	D Cuthbertson	D		Cuthbertson

	University of Liverpool	G Kemp	G		Kemp
	University of Liverpool	A McArdle	A	Anne	McArdle
	University of Liverpool	B Michael	B	Benedict	Michael
	University of Liverpool	W Reynolds	W	Will	Reynolds
	University of Liverpool	L G Spencer	L G	Lisa	Spencer
	University of Liverpool	B Vinson	B	Ben	Vinson
	University of Liverpool	M Ashworth	M		Ashworth
	University of Manchester	K Abel	K	Kathryn	Abel
	University of Manchester	H Chinoy	H		Chinoy
	University of Manchester	B Deakin	B	Bill	Deakin
	University of Manchester	M Harvie	M		Harvie
	University of Manchester	C A Miller	C A		Miller
	University of Manchester	S Stanel	S	Stefan	Stanel
	University of Manchester	P Barran	P	Perdita	Barran
	University of Manchester	D Trivedi	D	Drupad	Trivedi
	University of Newcastle	H McAllister-Williams	H	Hamish	McAllister-Williams
	University of Newcastle	S Paddick	S	Stella-Maria	Paddick
	University of Newcastle	A Rostron	A	Anthony	Rostron
	University of Newcastle	J P Taylor	J P	John Paul	Taylor
	University of Nottingham	D Baguley	D	David	Baguley
	University of Nottingham	C Coleman	C	Chris	Coleman
	University of Nottingham	E Cox	E		Cox
	University of Nottingham	L Fabbri	L	Laura	Fabbri
	University of Nottingham	S Francis	S	Susan	Francis
	University of Nottingham	I Hall	I	Ian	Hall
	University of Nottingham	E Hufton	E		Hufton
	University of Nottingham	S Johnson	S	Simon	Johnson

	University of Nottingham	F Khan	F	Fasih	Khan
	University of Nottingham	P Kitterick	P	Paaig	Kitterick
	University of Nottingham	R Morriss	R	Richard	Morriss
	University of Nottingham	N Selby	N	Nick	Selby
	University of Nottingham	L Wright	L	Louise	Wright
	University of Oxford	C Antoniades	C	Charalam bos	Antoniades
	University of Oxford	A Bates	A		Bates
	University of Oxford	M Beggs	M		Beggs
	University of Oxford	K Bhui	K	Kamaldee p	Bhui
	University of Oxford	K Breeze	K	Katie	Breeze
	University of Oxford	K M Channon	K M		Channon
	University of Oxford	D Clark	D	David	Clark
	University of Oxford	X Fu	X		Fu
	University of Oxford	M Husain	M	Masud	Husain
	University of Oxford	X Li	X		Li
	University of Oxford	E Lukaschuk	E		Lukaschuk
	University of Oxford	C McCracken	C	Celeste	McCracken
	University of Oxford	K McGlynn	K		McGlynn
	University of Oxford	R Menke	R		Menke
	University of Oxford	K Motohashi	K		Motohashi
	University of Oxford	T E Nichols	T E		Nichols
	University of Oxford	G Ogbole	G	Godwin	Ogbole
	University of Oxford	S Piechnik	S		Piechnik
	University of Oxford	I Propescu	I		Propescu
	University of Oxford	J Propescu	J		Propescu
	University of Oxford	A A Samat	A A		Samat
	University of Oxford	Z B Sanders	Z B		Sanders

	University of Oxford	L Sigfrid	L	Louise	Sigfrid
	University of Oxford	M Webster	M		Webster
	University of Oxford	L Kingham	L	Lucy	Kingham
	University of Oxford	P Klenerman	P	Paul	Klenerman
	University of Oxford	H Lamlum	H	Hanan	Lamlum
	University of Oxford & Oxford Health NHS Foundation Trust	M Taquet	M	Maxime	Taquet
	University of Oxford, Nuffield Department of Medicine	G Carson	G		Carson
	University of Sheffield	L Finnigan	L		Finnigan
	University of Sheffield	L C Saunders	L C	Laura	Saunders
	University of Sheffield	J M Wild	J M	James	Wild
	University of Southampton	P C Calder	P C		Calder
	University of Southampton	N Huneke	N	Nathan	Huneke
	University of Southampton	G Simons	G	Gemma	Simons
	University of Southampton	D Baldwin	D	David	Baldwin
	University of Swansea	S Bain	S	Steve	Bain
	Usher Institute, University of Edinburgh, Edinburgh, United Kingdom	L Daines	L	Luke	Daines
	Whittington Health NHS Trust	E Bright	E		Bright
	Whittington Health NHS Trust	P Crisp	P		Crisp
	Whittington Health NHS Trust	R Dharmagunawardena	R	Ruvini	Dharmagunawardena
	Whittington Health NHS Trust	M Stern	M		Stern
	Wirral University Teaching Hospital	L Bailey	L	Elisabeth	Bailey
	Wirral University Teaching Hospital	A Reddington	A	Anne	Reddington
	Wirral University Teaching Hospital	A Wight	A	Andrew	Wight
	Wrightington Wigan and Leigh NHS trust	A Ashish	A		Ashish
	Wrightington Wigan and Leigh NHS trust	J Cooper	J	Josh	Cooper
	Wrightington Wigan and Leigh NHS trust	E Robinson	E	Emma	Robinson
	Yeovil District Hospital NHS Foundation Trust	A Broadley	A	Andrew	Broadley

York & Scarborough NHS Foundation Trust	L Barman	L	Laura	Barman
York & Scarborough NHS Foundation Trust	C Brookes	C	Claire	Brookes
York & Scarborough NHS Foundation Trust	K Elliott	K		Elliott
York & Scarborough NHS Foundation Trust	L Griffiths	L		Griffiths
York & Scarborough NHS Foundation Trust	Z Guy	Z	Zoe	Guy
York & Scarborough NHS Foundation Trust	K Howard	K	Kate	Howard
York & Scarborough NHS Foundation Trust	D Ionita	D	Diana	Ionita
York & Scarborough NHS Foundation Trust	H Redfearn	H	Heidi	Redfearn
York & Scarborough NHS Foundation Trust	C Sarginson	C	Carol	Sarginson
York & Scarborough NHS Foundation Trust	A Turnbull	A	Alison	Turnbull
Newcastle upon Tyne Hospitals NHS Foundation Trust & University of Newcastle	R Penfold		Ruth	Penfold
Newcastle upon Tyne Hospitals NHS Foundation Trust & University of Newcastle	J Glennie		Jennifer	Glennie
Newcastle upon Tyne Hospitals NHS Foundation Trust & University of Newcastle	F Chambers		Francesca	Chambers
